# Traits and QTLs for development of dry direct-seeded rainfed rice varieties

**DOI:** 10.1093/jxb/eru413

**Published:** 2014-10-21

**Authors:** Nitika Sandhu, Rolando O. Torres, Ma. Teresa Sta Cruz, Paul Cornelio Maturan, Rajinder Jain, Arvind Kumar, Amelia Henry

**Affiliations:** ^1^Division of Plant Breeding, Genetics, and Biotechnology, International Rice Research Institute, DAPO Box 7777, Metro Manila, The Philippines; ^2^Department of Molecular Biology and Biotechnology, CCS Haryana Agricultural University, Hisar-125004, India; ^3^Crop and Environmental Sciences Division, International Rice Research Institute, DAPO Box 7777, Metro Manila, The Philippines

**Keywords:** Direct-seeded rice, early vegetative vigour, grain yield, nutrient uptake, quantitative trait loci (QTLs), root.

## Abstract

The development of rice varieties for dry direct-seeded conditions can be accelerated by selecting suitable traits. In the present investigation, traits hypothesized to be important for direct-seeded conditions in rainfed systems, including seedling emergence, early vegetative vigour, nutrient uptake, nodal root number, and root hair length and density, were characterized to study the genetic control of these traits and their relationship with grain yield under seedling- and reproductive-stage drought stress. Two BC_2_F_4_ mapping populations derived from crosses of Aus276, a drought-tolerant aus variety, with MTU1010 and IR64, high-yielding indica mega-varieties, were developed and studied to identify quantitative trait loci (QTLs) that showed large and consistent effects. A total of 26 QTLs associated with 23 traits and 20 QTLs associated with 13 traits were mapped in the Aus276/3*IR64 and Aus276/3*MTU1010 populations, respectively. qGY_6.1_, qGY_10.1_, qGY_1.1_, and qEVV_9.1_ were found to be effective in both populations under a wide range of conditions. QTLs for several seedling-stage traits co-located with QTLs for grain yield, including early vegetative vigour and root hair length. On chromosome 5, several QTLs for nutrient uptake co-located with QTLs for root hair density and nematode gall rating. Six lines were selected from both populations based on grain yield and the presence of QTLs, and these lines typically showed improved seedling-stage traits (nodal root number, dry shoot weight, and root hair length and density). The co-located QTLs identified here can be used in research aimed at increasing the yield and adaptability of rainfed rice to direct-seeded conditions.

## Highlight text

Characterization and QTL identification of seedling-stage traits revealed relationships with nutrient uptake and grain yield; these traits may improve the adaptation and productivity of rice under direct-seeded conditions.

## Introduction

The production of conventional puddled transplanted rice faces severe constraints because of water and labour scarcity, and climatic changes (Pathak *et al.*, 2011). Direct-seeded rice is a feasible alternative to conventional puddled transplanted rice with good potential for saving water, reducing labour requirements, mitigating greenhouse gas emissions, and adapting to climatic risks; and the yield can be comparable with that of transplanted rice if the crop is properly managed (Kumar and Ladha, 2011). Although the development of suitable varieties and agronomic packages for promoting direct-seeded rice is under way (Pathak *et al.*, 2011), so far no variety has been developed that possesses traits specifically needed to produce high yield under dry direct-seeded conditions, particularly for rainfed systems that may be prone to drought and low fertility.

Although dry direct-seeded rice fields may experience soil moisture regimes that range from aerobic to flooded after establishment, direct-seeded rice conditions are generally more favourable than transplanted conditions for the growth of weeds that compete with rice for nutrients, moisture, and sunlight, and can cause large yield losses (Pathak *et al.*, 2011). To improve initial crop establishment and the competitiveness of direct-seeded rice, varieties with higher germination and faster seedling emergence with more vigorous seedlings under dry direct-seeded conditions must be selected to minimize the risks encountered in direct seeding (Azhiri-Sigari *et al.*, 2005). During the vegetative stage, rapid ground cover achieved with early vegetative vigour (Poorter and De Jong, 1999; Shipley, 2006; Dingkuhn *et al.*, 1999) can reduce soil evaporation and accelerate root access to soil water and nutrients (Zhao *et al.*, 2006). Early vegetative vigour can be defined as a high relative growth rate (RGR) during exponential growth before canopy closure (Dingkuhn *et al.*, 1999; Poorter and De Jong, 1999; Shipley, 2006). Early vegetative vigour depends on the assimilate source (light capture and photosynthetic rate) as well as the sink constituted by structural growth (leaf appearance rate, potential size, and tiller outgrowth). Early vegetative vigour may also accelerate the depletion of soil water reserves, making less water available for later crop stages (Zhang *et al.*, 2005). However, in dry direct-seeded environments, early vegetative vigour is associated with yield stability (Okami *et al.*, 2011). Differences in the early vegetative vigour of rice cultivars affecting crop establishment under direct-seeded conditions have previously been reported in Asia (Garrity *et al.*, 1992; Caton *et al.*, 2003), Latin America, and Africa (Fischer *et al.*, 1997; Johnson *et al.*, 1998; Dingkuhn *et al.*, 1999).

Agricultural production in the 21st century is predicted to be more limited because of lower availability and increased cost of water and nutrient resources (Lal, 2007). Reduced nutrient uptake, especially of nitrogen and phosphorus, has been the most important factor for lower yield in direct-seeded cultivation systems compared with flooded systems of rice cultivation (Kumar and Ladha, 2011), and is a particular concern in low-input rainfed systems. This emphasizes the urgency of improving the rice root system so that plants are able to capture nutrients more efficiently. Strategies of efficient nutrient acquisition include root morphology for exploring nutrients in soils through the growth of axial roots with shallow angles and more dispersed lateral roots (Lynch, 2011), and the development of longer lateral roots with more root hairs (Rose *et al.*, 2012).

Root traits are typically complex and controlled by many genes, each with a small genetic effect; such traits are typically controlled by quantitative trait loci (QTLs; Sharma *et al.*, 2011). Identifying genetic variation and QTLs for root traits can contribute to our understanding of their role in plant performance under direct-seeded conditions. Direct-seeded conditions decrease the number of nodal roots per internode and stimulate the elongation of nodal roots (Kondo *et al.*, 1999). Other traits that may be important for direct-seeded rice are root hair growth and lateral root growth. Phosphorus uptake is closely related to root hair length, and plants grown under phosphate-limiting conditions form longer root hairs (Bates and Lynch, 1996; Zhang *et al.*, 2003). Increased lateral root growth increased phosphorus uptake in common bean (Lynch and Van Beem, 1993) and maize (Zhu and Lynch, 2004), nitrogen uptake (Sen *et al.*, 2013) and yield under varying water and nutrient regimes (Hochholdinger and Tuberosa, 2009) in maize, and water uptake under drought in rice (Suralta et al., 2008, 2010).

A number of QTLs associated with nutrient uptake have been detected in rice (Ni *et al.*, 1998; Wissuwa *et al.*, 1998; Ming *et al.*, 2000), maize (Zhu *et al.*, 2005), wheat (Su *et al.*, 2006, 2009), common bean (Liao *et al.*, 2004; Yan *et al.*, 2004), and soybean (Li *et al.*, 2005a; Liang *et al.*, 2010). Nutrient-efficient plants produce higher yield per unit of nutrient applied or absorbed than other plants grown under similar agroecological conditions (Fageria *et al.*, 2008). Although it is often emphasized that enhancing nutrient-use efficiency is critical across the globe both to sustain yield in low-input agriculture and to reduce fertilizer inputs in high-input agriculture, it is unclear whether traits that enhance efficiency in low-input systems also confer efficiency in high-input systems (Rose *et al.*, 2012). Therefore, better understanding of root-related traits and how root-trait QTLs interact to affect soil resource acquisition across a range of environments will be important in breeding direct-seeded rice varieties. The overall aim of this study was to investigate genetic variation and identify QTLs with large and consistent effects for traits thought to be beneficial for direct-seeded rice: seedling emergence, early vegetative vigour, root morphology, nutrient uptake, drought tolerance, and grain yield. We hypothesized that some of the seedling-stage traits investigated might have strong enough effects on plant growth to be correlated with grain yield at harvest.

## Materials and methods

### Development of mapping populations

The parents used to develop mapping populations were Aus276 (donor parent), an early-maturing traditional drought-tolerance donor and aus pure line originating from Bangladesh; IR64, a drought-sensitive indica mega-variety widely cultivated in Asia due to its high-yielding ability, preferred quality traits, and acceptable tolerance of major biotic stresses; and MTU1010, a moderately drought-sensitive indica variety widely grown in India.

The populations used for this study consisted of backcross-derived lines (BC_2_F_4_) from crosses of Aus276/3*IR64 and Aus276/3*MTU1010. The populations were developed through crossing of Aus276 with IR64 or MTU1010, and the resulting hybrids (F_1_) were backcrossed twice to IR64 or MTU1010 to produce BC_2_F_1_ lines. 206 single BC_2_F_1_ plants from Aus276/3*IR64 and 116 single BC_2_F_1_ plants from Aus276/3*MTU1010 were grown in the F_1_ nursery in the 2010 wet season (W). Plants were advanced in the F_2_ nursery in the 2011 dry season (D) and single panicles were harvested from each plant. Two to three seeds per panicle were collected, bulked, and advanced in 2011W. Three hundred plants from each population were taken randomly and used for this study.

### Field experiments for direct-seeded rice traits and agronomic studies

Sixteen field experiments were conducted at the International Rice Research Institute (IRRI), Los Baños, Laguna, The Philippines (14°10’11.81”N, 121°15’39.22”E). In this study, the term ‘lowland’ refers to flooded, puddled, transplanted, and anaerobic conditions, and ‘direct-seeded upland’ refers to directly sown, non-puddled, non-flooded, aerobic conditions in levelled fields. Two types of experiments were conducted: those for the evaluation of direct-seeded rice traits in which seedling-stage stress was induced, and those for the evaluation of agronomic traits in which reproductive-stage stress was induced (Table 1). The field experiments on direct-seeded rice traits were carried out in a rainout shelter under direct-seeded conditions in the 2012 wet season (2012W) and the 2013 dry season (2013D). The field experiment for the study of agronomic traits was carried out under lowland and upland conditions in 2012D and 2013D, and a well-watered control treatment (non-stress) was included each season. All 300 BC_2_F_4_ lines were included in each experiment.

**Table 1.** Details of the experiments conducted in 2012–2013 with their corresponding growing environment

Experiment	Condition	Environment code^a^	Stage of drought stress	Date of planting/ transplanting	Population	Observed traits^b^
1	Direct-seeded upland	2012DUR	Reproductive	11 January 2012	Aus276/3*IR64	PH, DTF, GY
2	Direct-seeded upland	2013DUR	Reproductive	22 January 2013	Aus276/3*IR64	EVV, PH, DTF, GY, biomass, HI
3	Direct-seeded upland	2012DUN	Non-stress	3 January 2012	Aus276/3*IR64	PH, DTF, GY
4	Direct-seeded upland	2013DUN	Non-stress	20 December 2013	Aus276/3*IR64	PH, DTF, GY
5	Direct-seeded upland	2012WUS	Seedling	20 June 2012	Aus276/3*IR64	EVV, NR, RHL, RHD, P concentration and uptake (seedling stage), PH, DTF, GY, biomass, HI
6	Direct-seeded upland	2013DUS	Seedling	17 January 2013	Aus276/3*IR64	EVV, NR, RHL, RHD, lateral root rating, nematode gall rating, P concentration and uptake (seedling stage), nutrient concentration and uptake [Al, Ca, Cu, Fe, K, Mg, N, Mn, Na, P, S, Zn (reproductive stage)], PH, DTF, GY, biomass, HI
7	Direct-seeded upland	2012DUR	Reproductive	11 January 2012	Aus276/3*MTU1010	PH, DTF, GY
8	Direct-seeded upland	2013DUR	Reproductive	22 January 2013	Aus276/3*MTU1010	EVV, PH, DTF, GY, biomass, HI
9	Direct-seeded upland	2012DUN	Non-stress	3 January 2012	Aus276/3*MTU1010	PH, DTF, GY
10	Direct-seeded upland	2013DUN	Non-stress	20 December 2013	Aus276/3*MTU1010	PH, DTF, GY
11	Direct-seeded upland	2012WUS	Seedling	20 June 2012	Aus276/3*MTU1010	EVV, NR, RHL, RHD, P concentration and uptake, PH, DTF, GY, biomass, HI
12	Direct-seeded upland	2013DUS	Seedling	17 January 2013	Aus276/3*MTU1010	EVV, NR, RHL, RHD, P concentration and uptake, PH, DTF, GY, biomass, HI
13	Transplanted lowland	2012DLR	Reproductive	20 December 2012/ 16 January 2012	Aus276/3*IR64	PH, DTF, GY
14	Transplanted lowland	2012DLN	Non-stress	10 December 2012/ 5 January 2012	Aus276/3*IR64	PH, DTF, GY
15	Transplanted lowland	2012DLR	Reproductive	20 December 2012/ 14 January 2012	Aus276/3*MTU1010	PH, DTF, GY
16	Transplanted lowland	2012DLN	Non-stress	10 December 2012/ 5 January 2012	Aus276/3*MTU1010	PH, DTF, GY

^a^ Environment codes: D, dry season; W, wet season; U, upland; L, lowland; N, non-stress (well-watered); R, reproductive-stage drought stress; S, seedling-stage drought stress.

^b^ PH, plant height; DTF, days to flowering; GY, grain yield; NR, number of nodal root; EVV, early vegetative vigour; RHL, root hair length; RHD, root hair density; HI, harvest index.

The field used for the seedling-stage stress experiments had been fallow for 5 years before the experiment was conducted. Soil characteristics included pH 7.7 (0.01M CaCl_2_); 16.0 meq 100g^–1^ Ca; 7.8 meq 100g^–1^ Mg; 1.09 meq 100g^–1^ K; 20mg kg^–1^ P; 0.101% Kj N; 39% clay; 21% sand; and 36% silt. A basal fertilizer was incorporated into the soil at a rate of 45-45-45kg ha^–1^ at 2 days after sowing (DAS). No topdressing was applied in order to maintain low-nutrient conditions representative of rainfed systems. Seeding was done at ~2-cm depth at 2g per linear meter in dry-ploughed plots randomized in an augmented design (CropStat version 7.2) with one replication of each genotype and two checks (Aus276 and IR64 or MTU1010) repeated ten times. Single-row plots (3 m) were planted with row spacing of 30cm. The same field area was used for both populations, but they were randomized separately (one population was on the left side of the field and the other was on the right side). Neighbouring plots served as borders, and border rows were planted on the edges of the experiments. Data on seedling emergence were noted from 2 DAS until 12 DAS in 2013DUS (see Table 1 for an explanation of environment codes). The central 1 m of each plot was thinned to an interplant spacing of ~5cm and maintained undisturbed to record observations for grain yield and yield-attributing traits, and the remaining 2 m of each row was used for destructive sampling. Plants were systematically sampled from the same location in each plot (0.5 m from the edge). Irrigation was stopped once all plots had emerged, and stress was initiated by closing the rainout shelter at 9 DAS until the experiments were re-watered (31 DAS) and kept well watered until maturity. Soil water potential was measured by tensiometers (15- and 30-cm soil depth) until crop maturity (Supplementary Figure S1).

Experiments for agronomic traits under lowland and direct-seeded upland conditions (Table 1) were planted in an alpha lattice design in one row by 2 m plots with two replications of each BC_2_F_4_ line. Each block included six randomized entries with single-row plots of 2 m with border rows planted on the edges of each block. Two checks (Aus276 and MTU1010 or IR64) were included 10 times in each replication in experiments 2012DUR, 2013DUR, 2012DUN, 2013DUN, 2012DLN, and 2012DLR. For all lowland trials, seeds were sown in a raised-bed nursery and 26-day-old seedlings were transplanted to the main field with each hill containing one seedling. For direct-seeded upland trials, seeds were sown directly into the soil with a row spacing of 0.3 and 0.25 m in all experiments in 2012 and 2013, respectively, using a seeding density of 2g per linear meter of row, resulting in a seed rate of ~305 seeds m^−2^ in 2012 and 365 seeds m^−2^ in 2013 (which also reflected slightly lower germination rates in 2012). The non-stress treatments in the lowland trials were maintained flooded and the direct-seeded upland trials were sprinkler-irrigated twice weekly. Stress treatments were irrigated during establishment and early vegetative growth, but irrigation was stopped in the reproductive-stage drought stress treatments at 30 days after transplanting (DAT)/51 DAS in the 2012 lowland trial, and at 56 and 40 DAS in 2012 and 2013, respectively, in the direct-seeded upland trials. Plots were re-irrigated periodically when most lines were wilted and exhibited leaf drying. Soil water potential was measured by tensiometers (30-cm soil depth) until crop maturity (Supplementary Figure S2).

### Characterization of seedling-stage and agronomic traits for direct-seeded rice

#### Emergence and early vegetative vigour 

Seedling emergence (first and full emergence) was recorded only in 2013DUS. Seedlings were sampled three times, at 15, 22, and 30 DAS, in 2012WUS and 2013DUS. Three plants per plot were removed from the soil by digging a hole with a trowel (10-cm depth), and the roots gently washed over a sieve. The numbers of nodal roots per plant were counted manually. The roots and shoots were then separated and shoots were dried in an oven at 60°C for 3 days.

In 2012WUS and 2013DUS, early vegetative vigour was calculated in terms of RGR (determined by shoot biomass accumulation):

$$\left(\frac{\ln\left(\text{dry shoot weight at sampling 2}\right)-\ln\left(\text{dry shoot weight at sampling 1}\right)}{\left(\text{date of sampling 2}-\text{date of sampling 1}\right)}\right)$$

In the 2013DUR, early vegetative vigour was recorded on a 1−9 scale at 30 DAS according to the IRRI Standard Evaluation System for rice (IRRI, 1996).

#### Characterization of root hair length and density 

In 2012WUS and 2013DUS, the roots from the third sampling (30 DAS) were stored in 70% ethanol. Root hairs on the basal 2−3cm of nodal roots were visually evaluated as described by Vieira *et al.* (2007) by two to three researchers, each observing different roots, after staining with 0.05% trypan blue, and using a rating scale of 1−5 to rank root hair length and density (Supplementary Table S1). Lateral roots were visually evaluated using a rating scale of 0−3 (Supplementary Table S1) in 2013DUS. Root hair ratings were given a combined score for length and density that were later evaluated separately, while the lateral roots were given one overall score (Supplementary Table S1).

#### Nutrient analysis at seedling stage and reproductive stage 

In 2012WUS and 2013DUS, a total of 100 lines from both populations covering a range of ratings for root hair length and density were selected. The shoots from three plants per plot at 22 DAS from both populations were sampled and dried at 70°C. Shoot samples were ground and ashed at 600°C for 16h. The ash was dissolved in 0.1 N HCl, and phosphorus concentration was then determined using a colourimetric assay by spectrophotometry (Murphy and Riley, 1962). At 95 DAS, the same above-mentioned 100 lines from the Aus276/3*IR64 population were chosen for nutrient analysis in 2013DUS. Shoots from three plants per plot were dried at 70°C, ground, and analysed by inductively coupled optical-emission spectrometry in the IRRI Analytical Service Laboratory. Nutrient uptake was calculated as concentration × biomass.

#### Nematode study 

During the root hair rating, we observed that the densest root hairs were found on parts of the nodal root that were swollen, which a preliminary evaluation suggested might be associated with nematode infection. Therefore, at 95 DAS, the above-mentioned 100 lines from the Aus276/3*IR64 population were chosen for quantifying nematode infection in 2013DUS under conditions of natural nematode occurrence. The roots of the three plants per plot (20-cm depth, sampled with a shovel) were used for nematode assays. Nematode galls per plant were visually evaluated using a rating scale of 0−5 according to Hussey and Janssen (2002).

#### Spatial mapping of soil and plant parameters 

A map of apparent soil electrical conductivity (EC) to a depth of 0.75cm was made between 2012W and 2013D when there was no standing crop (EM38, Geonics Inc.; Leica GPS) according to Klassen *et al.* (2014). GPS points were also acquired at each sampling point at 95 DAS in 2013DUS, when shoot and root samples for nematode gall rating were collected. Spatial heat maps for nematode gall rating, root hair length, density, phosphorus uptake, dry shoot weight, and grain yield (based on the position of each plot) were prepared. Spatial data were modelled in R (version 3.0.1, package: raster) using the thin plate spline regression function (*Tps*) and converted to a raster for plotting the heat maps using the predict function (*predict*). To evaluate relationships among the different soil and plant parameters, the data were spatially grouped into row-level plots (3.2 m × 0.25 m) within the experimental field using ArcGIS (ArcMap 10, ESRI). Mean plot data were then analysed by ‘correlation’ in R.

#### Characterization of agronomic traits 

Days to flowering (DTF) was recorded when 50% of the plants in the plot exserted their panicles in 2012DUN/DUR, 2013DUN/DUR, 2012DLN/DLN, 2012WUS, and 2013DUS. Plant height was measured as the mean height of five random plants for each entry measured from the base of the plant to the tip of the panicle during maturity stage. Above-ground biomass was sampled by cutting a 0.5-m length (near to the edge) of each single-row plot at ground level at maturity. This material was oven-dried, weighed, and subsequently used to determine biomass and harvest index (HI; grain yield/total aboveground biomass). The plants were harvested at physiological maturity or when 80−85% of the panicles turned to golden yellow and the panicles at the base were already at the hard dough stage; harvested grains were threshed and oven-dried for 3 days at 50°C. Moisture content was measured using a grain moisture meter, and grain weight data were normalized to a moisture content of 14% to determine grain yield (kg ha^−1^).

### Genotyping

#### DNA extraction and polymerase chain reaction 

DNA was extracted using the modified CTAB protocol (Murray and Thompson, 1980) from the fresh leaf samples collected from each entry of a single replication of 2012DUN in both mapping populations at 21 DAS and were lyophilized. The agarose gel electrophoresis method was used to check the quality and quantity of DNA with a reference λDNA. The DNA samples were diluted with 1 × TE into an equal concentration of 5ng µl^–1^ and were submitted to LGC Genomics Ltd (UK) for genotyping.

#### Polymorphism survey and whole-population genotyping 

Out of 1894 SNP markers, a total of 150 polymorphic SNP markers distributed on all 12 chromosomes at equal 10 cM for each population were selected for whole-population genotyping. Polymorphism surveying among the parents and whole-population genotyping was carried out by LGC Genomics Ltd. (UK).

#### Data analysis 

Data were analysed using Statistical Analysis System (SAS) v9.1.3 and CropStat v7.2.2007.2 (IRRI, 2007). Analysis of variance (ANOVA) was performed for each trait. Correlation analysis was also performed among the traits in each trial. Means of the lines were estimated using a linear mixed model in CropStat, considering replications and blocks within replications as random effects and lines as fixed effects.

Broad-sense heritability (H) was estimated as:

$$H=\frac{\sigma^{2}{}_{G}}{\sigma^{\text{2}}{}_{\text{G}}+(\sigma^{\text{2}}{}_{\text{E}}/r)}$$

where *σ* ^*2*^_*G*_ is the genotypic variance, *σ* ^*2*^_*E*_ is the error variance, and *r* is the number of replications.

#### QTL analysis and linkage map construction 

Three QTL software programs were used due to the availability of different analysis tools in each program and in order to focus on evaluation of only the most stable QTLs that were consistently identified in multiple programs. A locus was declared only if it was detected by all three software programs. Data for all traits measured (45 different traits under 16 different studies) were subject to QTL analysis using Windows QTL Cartographer version 2.5 (Wang *et al.*, 2011), and the analysis was subsequently repeated using QGene (Nelson, 1997) and QTL Network (Yang *et al.*, 2007). Phenotypic variance, flanking markers, position, and logarithm of the odds (LOD) score of the QTLs was calculated using Windows QTL Cartographer version 2.5 and also confirmed with Q Gene software (Nelson, 1997). The position of the QTLs was calculated as the mean of the positions detected across seasons and used to generate the chromosome maps in QTL Cartographer version 2.5 (Wang *et al.*, 2011). The additive effect as an absolute value varied with differences in severity of stress and did not reflect a proper estimation of the effect in the case of very severe drought stress (low additive effect absolute value) compared with mild drought stress (high additive effect absolute value). To correct this, we represented the additive effect as a percentage of the trial mean, which was calculated as:

AE%=AE×100/PM

where AE% is the percentage additive effect, AE is the nominal additive effect, and PM is the population mean. AE, PM, and the additive × additive interaction (in the case of epistatic QTLs) were calculated using QTL Network.

## Results

### Identification of QTLs for seedling-stage and agronomic traits for direct-seeded rice

**Fig. 1.**
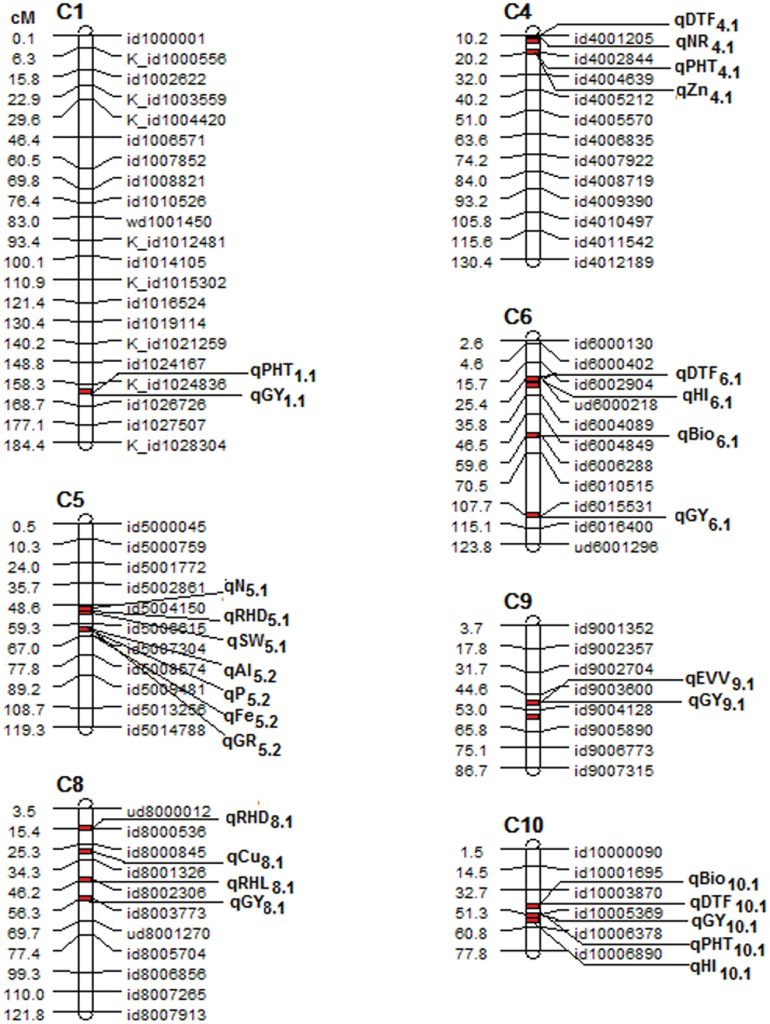
Chromosome map of QTLs detected across 16 studies under seedling-stage stress, reproductive-stage stress, and well-watered conditions in lowland and direct-seeded upland trials in the Aus276/3*IR64 population.

**Fig. 2.**
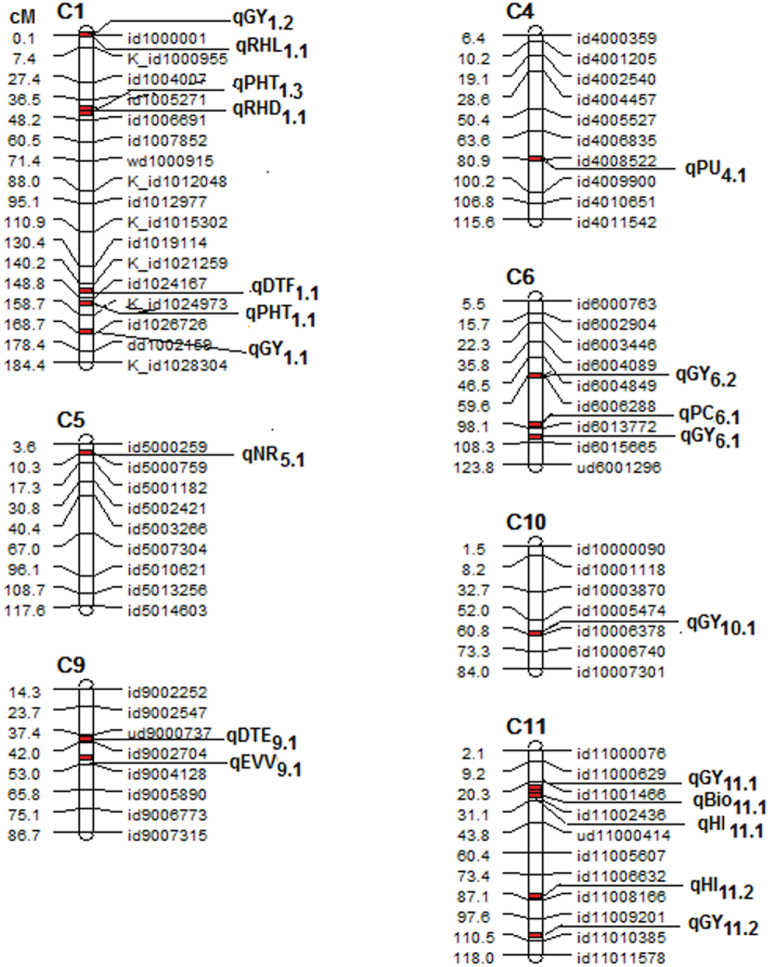
Chromosome map of QTLs detected across 16 studies under seedling-stage stress, reproductive-stage stress, and well-watered conditions in lowland and direct-seeded upland trials in the Aus276/3*MTU1010 population.

A wide range of phenotypic responses was observed among the BC_2_F_4_ lines in the seedling-stage stress trials for direct-seeded rice traits and in the reproductive-stage stress trials for agronomic traits (Supplementary Tables S2 and S3). A total of 26 QTLs associated with 23 traits were mapped using composite interval mapping (CIM) on chromosomes 1, 4, 5, 6, 8, 9, and 10 in the Aus276/3*IR64 population (Fig. 1; Supplementary Table S4), and 20 QTLs associated with 13 traits were mapped on chromosomes 1, 2, 4, 6, 8, 9, 10, and 11 in the Aus276/3*MTU1010 population (Fig. 2; Supplementary Table S5). Most QTLs identified in both populations were contributed by Aus276. The consistent effect of QTLs under different cultivation conditions over 2 years is supported by the significant *F* statistic values (*P* ≤ 0.01).

For agronomic traits, grain yield QTLs qGY_10.1_, qGY_6.1_, and qGY_1.1_ were identified to be stable in both populations over both years and in multiple conditions (Figs 1 and 2; Supplementary Tables S4 and S5). In most cases, the increase in grain yield can be attributed to the donor parent Aus276 (Supplementary Tables S4 and S5). However, in some cases, a negative value of the additive effect for the grain yield QTL can be attributed to the recipient parent (MTU1010/IR64), as reflected by the grain yield improvement in lines with and without the grain yield donor allele (Supplementary Tables S6 and S7). For time to flowering, qDTF_6.1_ and qDTF_10.1_ were identified, of which qDTF_10.1_ was co-located with qGY_10.1_ in both years in multiple conditions in the Aus276/3*IR64 population (Supplementary Table S4). For plant height, qPHT_1.1_ was mapped in both populations in both years in multiple conditions and was co-located with qGY_1.1_ under non-stress in the Aus276/3*IR64 population and located near qGY_1.1_ under non-stress in the Aus276/3*MTU1010 population (Supplementary Tables S4 and S5), and qPHT_10.1_ was co-located with qGY_10.1_ and qDTF_10.1_ on chromosome 10 under lowland stress and non-stress conditions in 2012 (Supplementary Table S4). In the Aus276/3*IR64 population, QTLs for DTF, HI, biomass, and grain yield were located within a region of ~11 cM on chromosome 10 (Supplementary Table S4). Similarly, QTLs for biomass, HI and grain yield were co-located in the Aus276/3*MTU1010 population on chromosome 11 (Supplementary Table S5).

**Fig. 3.**
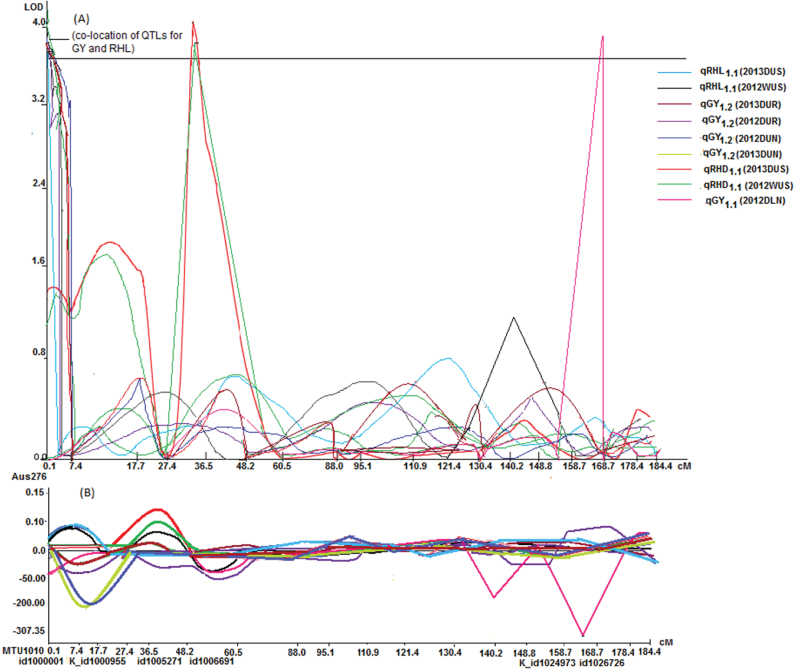
QTL likelihood curves of LOD scores with additive effects of coexisting grain yield, and root hair length and density QTLs. Regions shown are where QTLs for grain yield and root traits (RHL and RHD) hypothesized to be beneficial for direct-seeded conditions were co-located with QTLs for grain yield. (A) LOD score, and (B) additive effect in the Aus276/3*MTU1010 population on chromosome 1. Additive effect is the effect of substituting an Aus276 allele for a MTU1010 allele; a positive value indicates that Aus276 contributed the allele. (See Supplementary Table S5 for additive effect values).

**Fig. 4.**
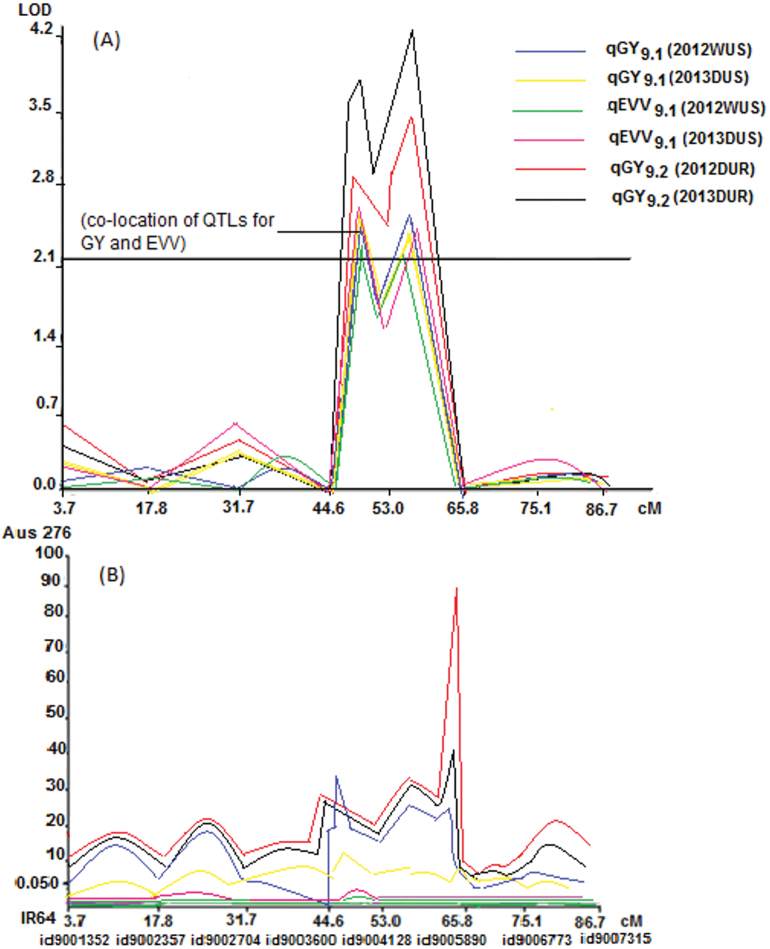
QTL likelihood curves of LOD scores with additive effects of coexisting QTLs. Regions shown are where QTLs for the early vegetative vigour trait hypothesized to be beneficial for direct-seeded conditions were co-located with QTLs for grain yield: (A) LOD score, and (B) additive effect in the Aus276/3*IR64 population on chromosome 9. Additive effect is the effect of substituting an Aus276 allele for an IR64 allele; a positive value indicates that Aus276 contributed the allele. (See Supplementary Table S4 for additive effect values).

**Fig. 5.**
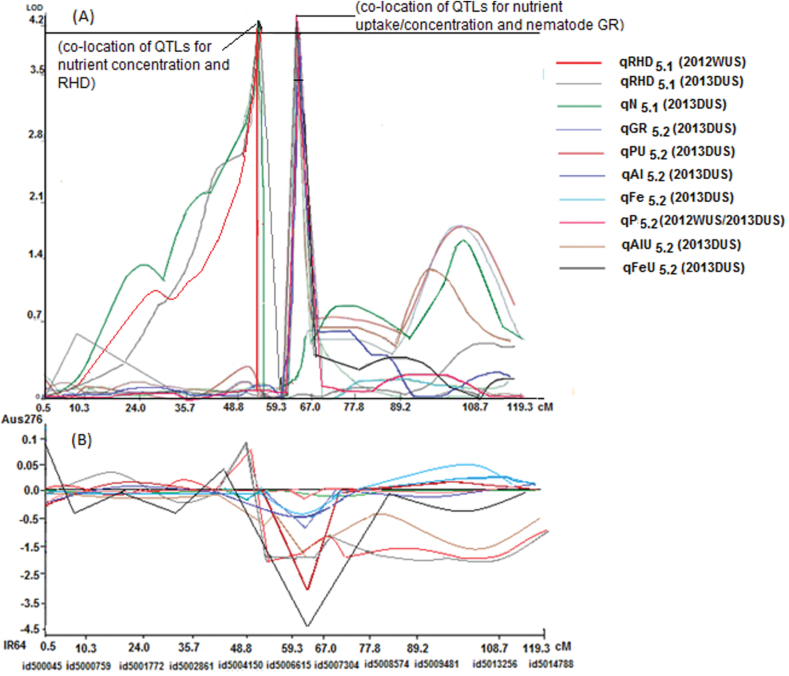
QTL likelihood curves of (A) LOD score (B) additive effect of coexisting QTLs for Al, Fe, N, and P uptake/concentration, root hair density, and nematode gall rating on chromosome 5 in the Aus276/3*IR64 population. Additive effect is the effect of substituting an Aus276 allele for an IR64 allele; a positive value indicates that Aus276 contributed the allele. (See Supplementary Table S4 for additive effect values).

A number of QTLs were also detected for traits hypothesized to be beneficial for direct-seeded conditions. The root hair density QTL qRHD_5.1_ was detected and co-located with qSW_5.1_ for seed weight in the Aus276/3*IR64 population (Supplementary Table S4). The root hair length QTL qRHL_1.1_ was co-located with qGY_1.2_ for grain yield in the Aus276/3*MTU1010 population (Fig. 3; Supplementary Table S5). qEVV_9.1_ for early vegetative vigour was mapped in both populations in multiple seasons under direct-seeded upland conditions and was co-located with qGY_9.1_ for grain yield (Figs 3 and 4). The majority of the QTLs identified for nutrient uptake (Al, Fe, and P) and nutrient concentration (Al, Fe, N, and P) were on chromosome 5 in the Aus276/3*IR64 population and this was also the region in which qGR_5.2_ for nematode gall rating was detected (Fig. 5). For leaf phosphorus concentration, qPC_6.1_ was located near qGY_6.2_ for grain yield under direct-seeded upland conditions in the Aus276/3*MTU1010 population.

The percentage increase in the different traits measured of lines possessing a donor allele compared with lines not possessing the allele was highest for root hair length (37.5−44.9%) in the Aus276/3*IR64 population and for grain yield (ranging up to 27%) in the Aus276/3*MTU1010 population. In addition to the major main-effect, single-locus QTLs for grain yield in the Aus276/3*IR64 mapping population, major epistatic QTLs for grain yield showing interaction between genomic locations on chromosomes 6 and 8 and between chromosomes 6 and 9 were identified (Supplementary Table S4; Supplementary Figure S3). Another pair of epistatic QTLs showing interaction between genomic locations on chromosomes 1 and 6 under direct-seeded upland conditions was identified for phosphorus uptake (Supplementary Table S5), which was co-located with qRHD_1.1_ and qGY_6.1_ in the Aus276/3*MTU1010 population.

### Characterization of traits for direct-seeded rice

The donor parent Aus276 showed higher seed weight, seedling emergence rates, early vegetative vigour, nutrient uptake, nodal root number, root hair length, and root hair density than the recipient parents IR64 and MTU1010, and this was reflected by higher mean values for these traits in the BC_2_F_4_ populations (Supplementary Table S2). In general, the QTLs for various traits showed a stronger phenotypic effect in the background of IR64 than in the background of MTU1010.

**Fig. 6.**
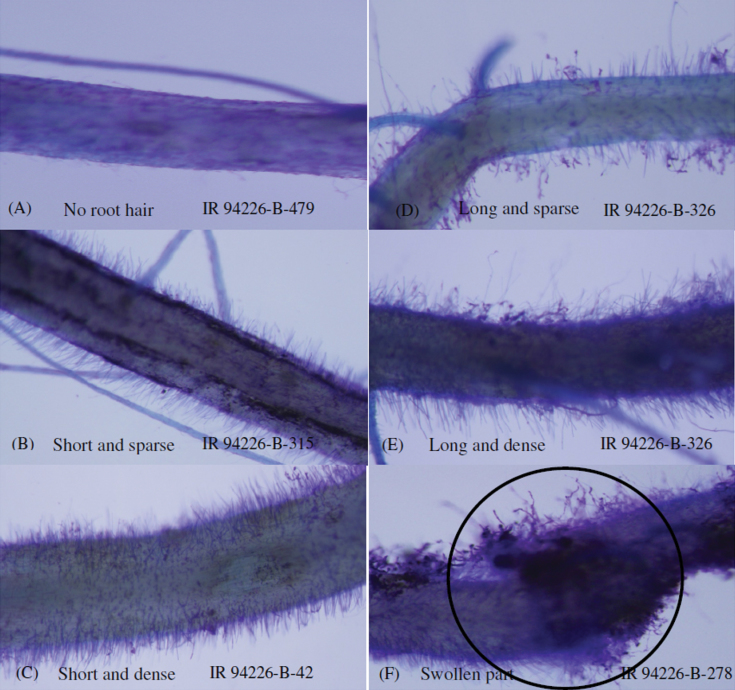
Variation in root hair length and density among lines under dry direct-seeded upland field conditions: (A−E) root hair rating (scale 1−5), (F) swollen root with dense root hair.

Root hairs were rated from field-grown roots (Fig. 6) and showed a range of densities, lengths, and overall morphologies; unlike agar-grown or paper rollup-grown root hairs, the root hairs from soil-grown roots were not always completely straight. In some samples, denser root hairs were observed in a swollen portion of the root (Fig. 6F), and preliminary evaluation indicated an interaction in the swollen portion with dense root hairs and nematode infection (Supplementary Figure S4). The interaction of dense root hair growth, nematode gall rating, and nutrient uptake was confirmed by the co-location of QTLs for these traits in the Aus276/3*IR64 population (Fig. 5).

The correlations among different seedling-stage and agronomic traits differed between populations (Table 2). Seed weight was negatively correlated with root hair length in both years in the Aus276/3*IR64 population, but not with other seedling-stage traits and not in the Aus276/3*MTU1010 population. In the Aus276/3*IR64 population, root hair density was positively correlated with grain yield in the seedling-stage stress treatment, and lateral root growth was correlated with grain yield and root hair density (Table 2). In the Aus276/3*MTU1010 population, early vegetative vigour (RGR) was correlated with grain yield in the direct-seeded, seedling-stage drought stress treatment, and nodal root number was correlated with early vegetative vigour, seedling emergence, and grain yield in the seedling-stage stress treatment (Table 2). Grain yield was negatively correlated with DTF in multiple environments, but it was positively correlated with DTF in the non-stress treatments of both populations in 2013D (Table 2). Biomass was significantly correlated with grain yield under direct-seeded conditions (Table 2). The heritability of grain yield was moderate in the upland trial while it was relatively high in the lowland trial (Table 3). The heritability of nodal root number, nutrient concentration, and uptake was moderate while it was low for root hair length and root hair density (Table 3; Supplementary Table S8).

**Table 2.** Correlation analyses among different seedling-stage traits (SSS experiment) and agronomic traits (DUR, WUS, DUS, and DUN experiments) of whole mapping populations derived from the Aus276/3*IR64 and Aus276/3*MTU1010 populations under reproductive and seedling stage drought upland direct-seeded conditions

Aus276/3*IR64 2012		**GY (DUR)** ^**a,b**^	**GY (WUS)**	**GY (DUN)**	**# NR (WUS)**	**EVV (WUS)**	**RHL (WUS)**	**RHD (WUS)**	**SW (WUS)**	**PHT (DUR)**	**DTF (DUR)**		
	GY (DUR)	1											
	GY (WUS)	0.1664*^c^	1										
	GY (DUN)	0.3179**	0.1943**	1									
	# NR	0.0806	0.0843	–0.0962	1								
	EVV	0.0614	0.0096	0.0023	0.1108	1							
	RHL	0.0562	0.0522	–0.0087	0.0603	0.0019	1						
	RHD	0.0586	0.1278*	0.001	0.0265	–0.0014	–0.1222	1					
	SW	0.1345*	0.0633	0.1564**	0.0781	0.002	–0.1033*	0.004	1				
	PHT	0.22**	0.162*	0.35**	–0.168**	–0.1340*	–0.0133	0.027	0.0773	1			
	DTF	–0.450**	–0.1930*	–0.61	–0.0392	0.0602	0.0461	0.0257	–0.2097**	–0.499**	1		
	Biomass	0.0355	0.7128**	0.1076	–0.1264	0.0508	0.0249	0.0756	0.0239	–0.1299	0.0869		
Aus276/3*IR64 2013		**GY (DUR)**	**GY (DUS)**	**GY (DUN)**	**# NR (DUS)**	**EVV (DUS)**	**RHL (DUS)**	**RHD (DUS)**	**Emergence (DUS)**	**SW (DUS)**	**LR (DUS)**	**PHT (DUR)**	**DTF (DUR)**
	GY (DUR)	1											
	GY (DUS)	0.4357*	1										
	GY (DUN)	0.1756**	0.193**	1									
	# NR	0.0096	0.0063	–0.0229	1								
	EVV	0.0316	0.0277	–0.0333	0.0195	1							
	RHL	0.0019	0.0222	–0.019	0.0011	0.0207	1						
	RHD	0.0623	0.1117*	0.0227	0.0739	–0.0163	–0.0669	1					
	Emergence	0.0343	0.0793	0.06	0.0117	0.1199*	0.0619	0.0198	1				
	SW	0.0606	0.0842	0.1253*	0.0211	0.0381	–0.1199*	–0.01	0.0606	1			
	LR	–0.019	0.1089*	–0.0285	–0.1125	–0.1095	–0.0217	0.1062*	–0.1469*	–0.0255	1		
	PHT	0.26*	0.2385*	0.48**	–0.185**	0.0598	–0.0395	0.1334*	0.0208	–0.0618	0.069	1	
	DTF	–0.65*	–0.248**	0.29*	–0.0646	0.1190*	0.0224	–0.1049	–0.0242	–0.196**	0.0736	–0.185**	1
	Biomass	0.031	0.1263*	0.0068	0.1496**	0.0879	0.0094	–120*	0.1224*	–0.0867	0.0225	0.0643	0.1034
		**GY (DUR)**	**GY (WUS)**	**GY (DUN)**	**# NR (WUS)**	**EVV (WUS)**	**RHL (WUS)**	**RHD (WUS)**	**SW (WUS)**	**PHT (DUR)**	**DTF (DUR)**		
Aus276/3*MTU1010 2012	**GY (DUR)**	1											
	**GY (WUS)**	0.0359	1										
	**GY (DUN)**	0.1720**	0.0187*	1									
	**# NR**	0.0387	0.1159	0.201**	1								
	**EVV**	0.036	0.0053	–0.0861	0.2272**	1							
	**RHL**	–0.0645	–0.0741	–0.1310*	0.0173	0.056	1						
	**RHD**	0.0233	0.0275	0.0903	0.0313	0.089	0.1347*	1					
	**SW**	0.1558*	0.061	0.0699	0.0293	–0.0205	0.0141	–0.0043	1				
	**PHT**	0.41*	0.239*	0.02	–0.0946	0.1273*	0.0623	–0.0467	0.1171	1			
	**DTF**	–0.32**	0.049	–0.47*	–0.0879	–0.193**	0.0596	0.0296	–0.2308**	–0.363**	1		
	**Biomass**	0.0265	0.5930**	0.0187	0.0288	0.1185	–0.0294	–0.0328	0.0416	–0.0389	0.049		
		**GY (DUR)**	**GY (DUS)**	**GY (DUN)**	**# NR (DUS)**	**EVV (DUS)**	**RHL (DUS)**	**RHD (DUS)**	**Emergence (DUS)**	**SW (DUS)**	**LR (DUS)**	**PHT (DUR)**	**DTF** **(DUR)**
Aus276/3*MTU1010 2013	**GY (DUR)**	1											
	**GY (WUS)**	0.0264	1										
	**GY (DUN)**	0.1756**	0.1930**	1									
	**# NR**	0.0682	0.1097*	0.0486	1								
	**EVV**	0.1827**	0.1238*	–0.0702	0.0630*	1							
	**RHL**	–0.0365	–0.0041	–0.0346	0.0747	0.1013	1						
	**RHD**	0.0762	0.0212	0.0253	–0.0399	0.1333*	0.1720**	1					
	**Emergence**	0.0159	0.0926	–0.153**	0.1959**	0.1304*	0.071	0.0324	1				
	**SW**	0.0759	0.0842	0.1121*	0.0211	0.0211	0.1011	–0.0221	0.0182	1			
	**PHT**	0.22**	0.1661	0.21*	–0.0463	0.0929	–0.0362	–0.0689	0.0739	0.1283	–0.1333	1	
	**DTF**	–0.14*	0.1323*	0.29*	–0.042	–0.0251	0.1246*	0.0455	–0.0525	–0.1711	0.1182	–0.307**	1
	**Biomass**	0.0814	0.2526**	0.1921	0.1163*	0.0158	–0.0672	–0.0304	0.1387*	0.0512	–	0.158	0.0574

^a^ Environment codes: U, upland; L, lowland; D, dry season; W, wet season; N, non-stress (well-watered); R, reproductive-stage drought stress; S, seedling-stage drought stress.

^b^ GY, grain yield; NR, nodal root number; RGR, relative growth rate; RHL, root hair length; RHD, root hair density; SW, seed weight; PHT, plant height; DTF, days to flowering; biomass, biomass at harvesting stage.

^c^ *, *P*<0.05; **, *P*<0.01.

**Table 3.** Broad-sense heritability of different traits under different experiments for Aus276/3*MTU1010 and Aus276/3*IR64 mapping populations for seedling-stage and agronomic traits^a^

Population	Traits^b^	Broad-sense heritability						
		2012WUS^c^	2013DUS	2012DUR	2013DUR	2012DUN	2012DLR	2012DLN
Aus276/3*MTU1010	DTF	0.26	0.40	0.17	0.38	0.50	0.42	0.71
	PHT	0.34	0.28	0.22	0.53	0.45	0.73	0.81
	GY	0.36	0.44	0.42	0.34	0.50	0.38	0.64
	Biomass	0.41	0.28	–	0.51	–	–	–
	HI	0.32	0.33	–	0.42	–	–	–
	NR	0.45	0.53	–	–	–	–	–
	EVV	0.39	0.26	–	0.22	–	–	–
	RHL	0.21	0.22	–	–	–	–	–
	RHD	0.18	0.23	–	–	–	–	–
	Seedling emergence	–	0.08	–	–	–	–	–
Aus276/3*IR64	DTF	0.42	0.35	0.26	0.43	0.49	0.56	0.85
	PHT	0.22	0.43	0.29	0.50	0.48	0.78	0.79
	GY	0.36	0.44	0.33	0.46	0.57	0.26	0.67
	Biomass	0.65	0.40	–	0.43	–	–	–
	HI	0.41	0.35	–	0.50	–	–	–
	NR	0.53	0.40	–	–	–	–	–
	EVV	0.44	0.30	–	0.26	–	–	–
	RHL	0.15	0.10	–	–	–	–	–
	RHD	0.09	0.14	–	–	–	–	–
	LRR	–	0.08	–	–	–	–	–
	Seedling emergence	–	0.12	–	–	–	–	–
	K concentration	–	0.53	–	–	–	–	–
	N concentration	–	0.74	–	–	–	–	–
	P concentration	–	0.23	–	–	–	–	–
	K uptake	–	0.32	–	–	–	–	–
	N uptake	–	0.33	–	–	–	–	–
	P uptake	–	0.28	–	–	–	–	–

^a^ Nutrient analysis conducted on plants sampled at 95 DAS.

^b^ GY, grain yield; NR, nodal root number; EVV, early vegetative vigour; RHL, root hair length; RHD, root hair density; LRR, lateral root rating; HI, harvesting index; SW, seed weight; PHT, plant height; DTF, days to flowering; biomass, biomass at harvesting stage.

^c^ Environment codes: U, upland; L, lowland; D, dry season; W, wet season; N, non-stress (well-watered); R, reproductive-stage drought stress; S, seedling-stage drought stress.

**Fig. 7.**
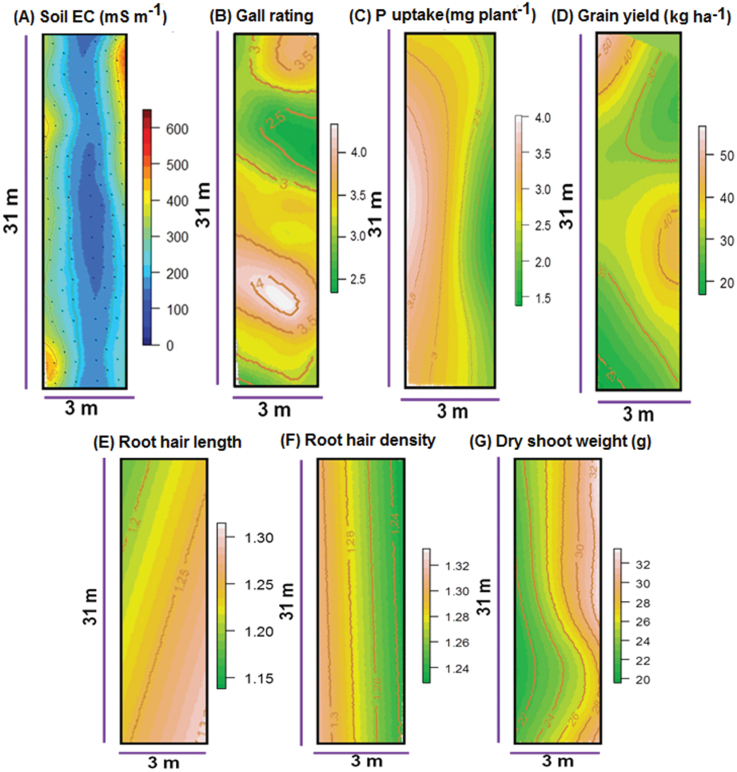
(A) Soil EC map and (B−G) spatial heat maps for gall rating (95 DAS), P uptake (22 DAS), grain yield (130 DAS), root hair length, root hair density (30 DAS), and dry shoot weight (95 DAS), in the 2013DUS trial in the Aus276/3*IR64 population.

To investigate the correlation between traits and the potential effects of spatial variation, a soil EC map and heat maps of the traits were generated; each showed distinct spatial variation patterns across the experimental field (Fig. 7), but grouping of each trait by plot revealed significant and positive correlation between soil EC and P uptake, whereas root hair length was found to be negatively correlated with P uptake (Table 4). These correlations were specific to the 100 selected lines that were plotted in the Aus276/3*IR64 population.

**Table 4.** Correlation matrix for the respective parameters that were compared in the soil maps from 2013DUS in 100 selected lines in the Aus276/3*IR64 population^a^

	Soil EC	Nematode gall rating	P uptake	GY	RHL	RHD	DSW
Soil EC	1						
Nematode gall rating	–0.07	1					
P uptake	0.37***	0.03	1				
GY	0.08	0.04	–0.22*	1			
RHL	–0.14	–0.09	–0.33***	–0.07	1		
RHD	0.07	0.08	–0.02	–0.04	0	1	
DSW	–0.41	–0.1	–0.33***	0.16	–0.04	–0.02	1

^a^ D, dry season; S, seedling-stage drought stress; EC, soil apparent electrical conductivity; P uptake, P uptake at seedling stage; GY, grain yield; RHL, root hair length; RHD, root hair density; DSW, dry shoot weight at seedling stage.

**Fig. 8.**
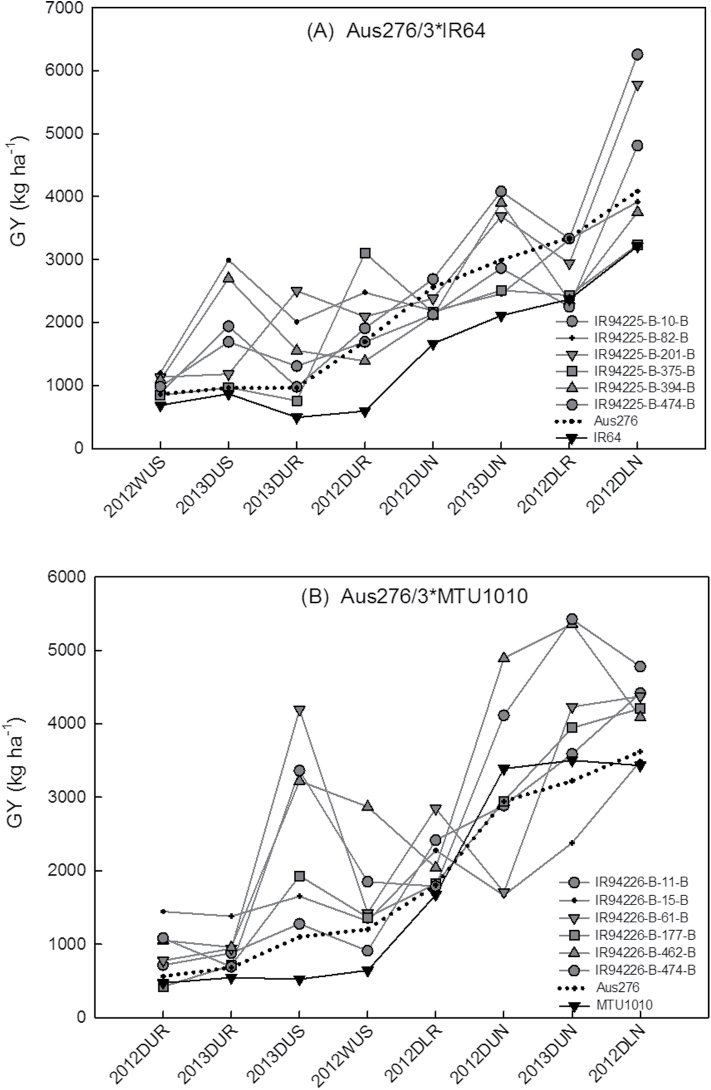
Response of six selected lines in terms of grain yield (GY) under different cultivation conditions: (A) Aus276/3*IR64 and (B) Aus276/3*MTU1010 populations. In the 2012WUS and 2013DUS experiments, each line was grown in one replicate in an augmented design. In the 2012DUR, 2013DUR, 2012DLR, and 2012DLN experiments, each line was grown in two replicates in an alpha lattice design.

**Fig. 9.**
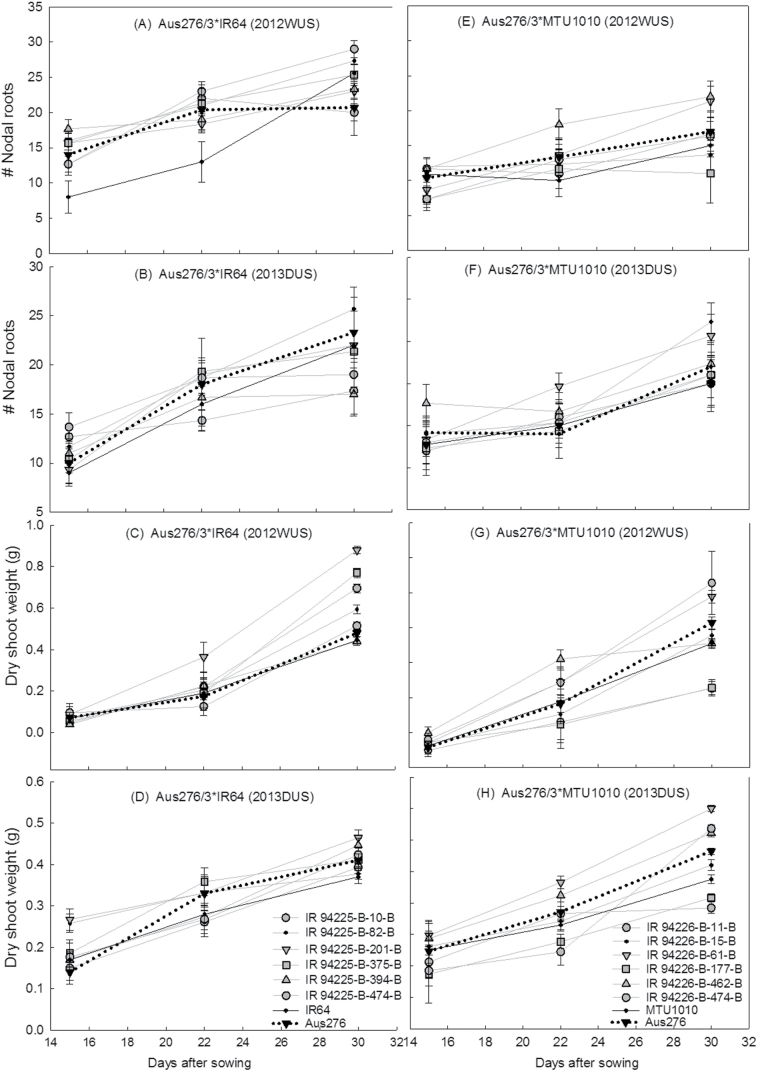
Response of six selected lines in terms of number of nodal roots and dry shoot weight (g) under dry direct-seeded upland conditions in the Aus276/3*IR64 (A−D) and Aus276/3*MTU1010 (E−H) populations. Each BC_2_F_4_ line was grown in one replicate in an augmented design; each point is the mean ± SE of three subreplicate plants sampled for each measurement.

**Fig. 10.**
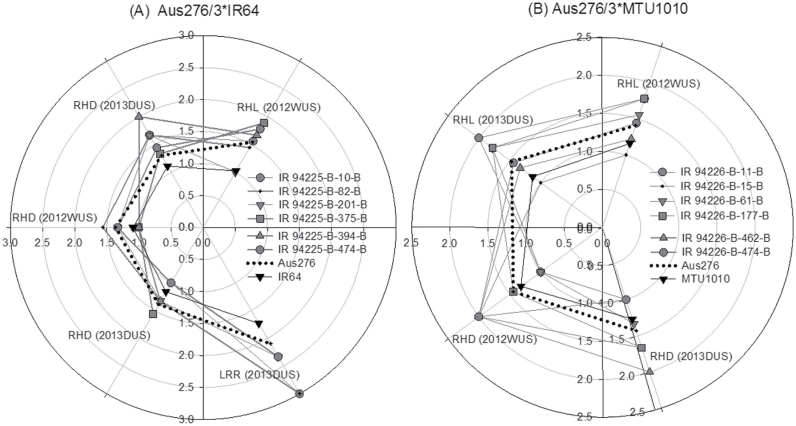
Response of six selected lines in terms of root hair length (RHL), root hair density (RHD), and lateral root rating (LRR) under dry direct-seeded upland conditions in the Aus276/3*IR64 (A) and Aus276/3*MTU1010 (B) populations. Each line was grown in one replicate in an augmented design.

From each population, BC_2_F_4_ lines were selected in which the most stable grain yield QTLs were present, as well as QTLs for early vegetative vigour, nodal root number, root hair density, and root length density. Of those lines with the grain yield and direct-seeded rice trait QTLs, six lines were identified that showed the most stable and highest yield in both years and in multiple conditions (Supplementary Table S9; Fig. 8). Compared with the parents (IR64 and MTU1010), many (but not all) of these selected lines showed higher nodal root numbers and higher dry shoot biomass related to early vegetative vigour throughout the seedling stage (Fig. 9). Likewise, many but not all of the selected lines showed longer and denser root hairs and higher lateral root rating than the parents (Fig. 10).

## Discussion

Direct relationships between seedling-stage traits and grain yield were established in terms of QTL co-location and phenotypic correlation. The co-location of QTLs for grain yield with root hair length on chromosome 9 and early vegetative vigour on chromosome 1, as well as the concentration of QTLs for nutrient uptake and concentration and root hair density on chromosome 5, implies that these could be key genetic regions for improving rice for direct-seeded conditions. The significant and positive correlations between some root traits (root hair density, and lateral root and nodal root number) and early vegetative vigour and yield under dry direct-seeded conditions indicate the role of improved water and nutrient uptake at the seedling stage in improving rice yield for direct-seeded conditions.

Interestingly, QTLs for root hair length and grain yield and for root hair density and phosphorus uptake were found to be co-located with each other in both populations. Compared with flooded paddy soils, reduced nutrient uptake, especially of nitrogen and phosphorus, under dry direct-seeded conditions has been the most important factor for lower yield in direct-seeded rice systems (Alam, 1999). The characterization of root-related traits and QTLs associated with these root traits (Sandhu *et al.*, 2013) that increase nutrient uptake under direct-seeded conditions can help develop direct-seeded rice varieties with high yield potential. The co-location of QTLs for grain yield with regions identified to govern seedling emergence, early vegetative vigour, root hair length, DTF, plant height, biomass, or HI indicates that some of these traits may be linked to each other, which may be useful for adaptation to direct seeding. Drought tolerance donors such as Aus276 have been categorized in the aus subgroup (Glaszmann, 1987), which is a pool of traditional varieties commonly used as donors for abiotic stress tolerance traits, and these varieties may have originally been cultivated in dry direct-seeded conditions. The increased values in seedling-stage dry shoot weight, nodal root number, lateral root growth, and root hair growth in the six selected lines that were chosen based on yield and presence of QTLs (Figs 9 and 10) further supports the importance of seedling-stage traits on grain yield under direct-seeded conditions. However, the selected lines did not consistently stand out as superior for all seedling traits, and the correlation coefficients between root traits and grain yield (Table 2) were low; these results are expected because yield is a function of many traits in addition to root traits. We found it interesting that the correlations observed between root traits and grain yield all showed the same patterns of correlation (+ or –) across both seasons and genetic backgrounds.

QTLs for phosphorus uptake in rice have been reported on chromosomes 2, 6, 10, and 12 (Wissuwa *et al.*, 2002). Although QTLs for root hair density and phosphorus uptake were co-located in this study on chromosome 5, no direct significant correlation between phosphorus uptake at the seedling stage or reproductive stage and root hair density was observed, and root hair length was negatively correlated with P uptake at seedling stage (Table 4). It can be hypothesized that there was a strong G × E interaction among seedling-stage traits that affected their direct correlation, as revealed by the correlations from the soil maps (Fig. 7; Table 4). Furthermore, the single-row layout in the SSS experiments may have resulted in inter-row competition that influenced the genotypic effects. Although these experiments were not conducted under P-deficient conditions (soil P-Olsen = 20mg kg^–1^), low-input conditions were imposed by withholding fertilizer application. Root hairs tend to be sparse in P-sufficient plants (Gahoonia and Nielsen, 1997). Therefore, it may be possible that under low-input conditions some of the lines became P deficient, and in response formed longer or denser root hairs as observed here (Table 4). Such a response is also supported by the negative relationship between root hair length and seed weight observed in the Aus276/3*IR64 population; smaller seeds may have supplied less resources to the growing seedlings, which in turn grew longer root hairs. It is also likely that the complex nature of root growth accounts for the lack of a direct relationship among traits (Table 2). Root architecture encompasses many interacting traits and components within a trait. Root hair growth was evaluated here only at the basal end of the nodal roots and it may differ in different parts of the root zone. Rice lateral roots fall into several different classes (Yamauchi et al., 1987a,b), can be highly affected by fluctuating conditions throughout a growing season (Suralta et al., 2008, 2010), and were evaluated here only by an overall rating at the basal end of the nodal roots. The number of nodal roots varied and originated from varying numbers of nodes; although this trait was not quantified in our study, it may be important for soil resource acquisition (Lynch and Brown, 2012) and should be further investigated. All of these traits probably interacted to affect phosphorus uptake in this study.

The co-location of QTLs on chromosome 5 in the Aus276/3*IR64 population indicates an interaction between nematode infection and root hair growth, which may have subsequently affected nutrient uptake. Although nematode infection was not significantly correlated with the other traits measured (Table 4), nematode infection possibly led to a change in root hair density that increased the ability to take up nutrients under dry direct-seeded conditions. A positive effect of root-knot nematodes on root hair growth, root exudation, and nutrient uptake has been reported in barley (Haase *et al.*, 2007). However, nematode infection is a serious concern for rice growing in aerobic soils, including dry direct-seeded conditions (Pankaj *et al.*, 2012). In this study in which only plant responses to naturally occuring nematode levels were evaluated, no notable damage to the crop was observed. Therefore the interaction of nematode infection with root hair density observed here should be further characterized under controlled levels of nematode application to identify any potential trade-offs in selecting for traits for direct-seeded rice.

The co-location of QTLs inherited from different parents, such as qRHD_5.1_ and QTLs for nutrient concentrations/uptake, suggest that it may be difficult to combine both of these QTLs in a MAS programme. It will be necessary to fine map the particular region, which may lead to the splitting of the region contributed by different parents. This would facilitate the precise introgression of the QTLs without undesirable linkages.

The use of cultivars with early vegetative vigour facilitating good crop establishment and better competitive ability with weeds is an important strategy for enhancing and stabilizing the yield of dry direct-seeded rice. Though several high-yielding cultivars have been released for cultivation in dry direct-seeded upland conditions, few have been widely adopted by farmers due to a lack of either high seedling vigour or preferred grain traits (Sujay, 2007). In the present study, qEVV_9.1_ for early vegetative vigour was detected in both populations and co-located with qGY_9.1_ in the Aus276/3*IR64 population under dry direct-seeded conditions. QTLs previously reported for root length (Uga *et al.*, 2010; Sandhu *et al.*, 2013), root thickness, and root number (Li *et al.*, 2005b) were located near or coincided with qEVV_9.1_ and qGY_9.1_ in our study. Although co-location of physiological traits and grain yield has been reported previously (Venuprasad *et al.*, 2002; Hanamaratti, 2007; Gautami *et al.*, 2012), it is not often that seedling-stage traits are related to yield due to differences in the time of their occurrence and fluctuations in environmental conditions between those time points. A better understanding of the nature of association among seedling traits, namely, early vegetative vigour, nodal root number, root hair length and density, and grain yield, is expected to provide a new dimension in planning selection strategies for rice adapted to a range of conditions.

A wide range of conditions (upland, lowland, seedling-stage stress and low-input soil management, reproductive-stage stress, and non-stress) was used to evaluate the populations because any of these conditions could be experienced by farmers using direct seeding, and also in order to confirm whether the loci are associated with low QTL × environment interaction, which is one of the two major requirements for the use of a QTL in MAS. Large-effect QTLs for grain yield under drought have been identified in rice (Bernier *et al.*, 2007; Venuprasad *et al.*, 2009) and their successful introgression has established a yield advantage under drought (Swamy *et al.*, 2013).

For developing drought-tolerant and direct-seeded rice with high yield potential, three major QTLs with a large effect on grain yield under dry direct-seeded conditions across a wide range of environments and backgrounds identified in our study can also be incorporated into future QTL introgression work in efforts to improve rice yield under dry direct-seeded drought conditions. Several of the QTLs identified for grain yield in our study (qGY_1.1_, qGY_6.1_, qGY_8.1_, qGY_9.1_, and qGY_10.1_) were previously reported to be consistent across different mapping populations and under different drought severities (Dixit *et al.*, 2012; Ghimire *et al.*, 2012; Vikram *et al.*, 2012; Sandhu *et al.*, 2013). The occurrence of different QTLs despite a common donor parent being used to develop both populations (Aus276/3*IR64 and Aus276/3*MTU1010) may be due to interaction between the QTLs and the genetic background of the recipient parent or epistatic interaction (Li *et al.*, 2000; Holland, 2001). The negative value of additive effect for qGY_1.2_ indicates that the yield-increasing allele was derived from the susceptible parent. Similar results were reported by Bernier *et al.* (2007) in which the allele increasing the grain yield was from Way Rarem, a drought-susceptible parent. In general, more phenotypic variation was observed in the Aus276/3*IR64 population than in the Aus276/3*MTU1010 population.

In addition to the effects of QTLs at single loci, it has been hypothesized that epistasis is one component of the genetic basis of quantitative traits, and a large portion of the main-effect QTLs in rice is involved in epistatic interactions (Xing *et al.*, 2002). In our study, we observed epistatic interactions between several QTLs showing effects on different traits (Supplementary Table S4; Supplementary Figure S2). The positive interaction between the genomic regions for root traits and nutrient uptake indicates that these interacting loci should also be introgressed together to obtain maximum positive effects on grain yield under dry direct-seeded conditions.

Our study identified the co-location of QTLs for grain yield and DTF and QTLs for grain yield and plant height. qDTF_10.1_ was co-located with qGY_10.1_ in both years in multiple conditions in the Aus276/3*IR64 population. The lines possessing both qGY_10.1_ and qDTF_10.1_ showed yield improvement of 13, 5, and 12% under upland non-stress, lowland non-stress, and lowland stress conditions, respectively, with 8 and 9 days earliness in flowering under upland and lowland non-stress conditions, respectively (Supplementary Table S10). The co-location of QTLs for agronomic traits (DTF, biomass, HI, and grain yield) under upland and lowland non-stress and stress conditions indicates that the increased grain yield observed was a result of interaction among all these traits. Bernier *et al.* (2007) and Vikram et al (2011) also reported the contribution of increased biomass and HI, and reduced time to flowering, to an increase in grain yield under reproductive-stage drought stress. The significance of qDTF_10.1_ under non-stress and reproductive-stage lowland conditions, as well as the significant negative relationships between grain yield and DTF, showed that the increase in grain yield under drought stress may be partly attributed to drought escape.

For plant height, qPHT_1.1_ and qGY_1.1_ (under non-stress in the Aus276/3*IR64 population) as well as qPHT_10.1_ and qGY_10.1_ were co-located. qPHT_4.1_ for plant height showed an effect in 2012DUN and 2013DUN, and qPHT_4.2_ showed an effect in 2012DUR and 2013DUR (Supplementary Table S4), both of which were previously reported to be located near QTLs for biomass yield and plant height, respectively (Bernier *et al.*, 2007). Co-location of QTLs can be attributed to the effect of pleiotropy or very close linkage of genes. It may be possible that the variation caused by QTLs governing DTF and/or PHT located in these regions, in conjunction with the timing of stress imposed, caused their effects on grain yield. The co-location of grain yield and DTF QTLs provides a unique opportunity to use them in the background of traditionally long-duration highly favoured varieties to develop high-yielding varieties for dry direct seeding with reduced duration that are suitable for cultivation under the present scenario of reduced water availability. Similarly, the co-location of grain yield and plant height QTLs can be used to increase plant height from the present semi-dwarf level to further increase yield under dry direct-seeded conditions. However, the co-location and significant relationships between seedling-stage traits and grain yield, in addition to DTF, illustrate the complexity of factors contributing to increased grain yield under direct-seeded conditions.

The magnitude and direction of influence of co-located loci on the different phenotypes will markedly affect the utility of such loci in selection for simultaneous improvement of these traits (Hittalmani *et al.*, 2003). Some of the donor alleles in the QTL regions identified in the present study may show negative interactions. For example, qRHL_1.1_ and qGY_1.2_ showed additive effects in opposite directions; such alleles should be identified and removed before using the QTLs in a MAS breeding program. In such cases where a single allele affects two different traits in different ways, the trait that is more advantageous or correlated to grain yield should be chosen for MAS. In such cases, fine mapping may help in the introgression of the specific QTLs/donor alleles for the desired trait without undesirable linkages. Coexisting chromosomal regions/loci governing different traits provide a unique opportunity for breeders to introgress such regions together as a unit into high-yielding lowland varieties through marker-assisted backcrossing and to develop cultivars possessing increased adaptation to dry direct-seeded conditions. The QTLs identified in this study for root traits that may increase nutrient uptake may be useful for future QTL introgression work to improve rice yield under dry direct-seeded conditions. All of the selected lines from this study will serve as novel materials for the development of stable direct-seeded rice varieties. Development of these direct-seeded varieties could be of significant benefit to farmers in regions with unstable rainfall who depend on rice for food security. The challenges ahead are the effective use of these root and grain yield QTLs and their combinations in breeding for direct-seeded rice varieties, fine mapping of QTLs to facilitate precise introgression without undesirable linkages, evaluation of their effectiveness in other genetic backgrounds, and understanding the physiological and molecular mechanisms associated with these major-effect QTLs under direct-seeded conditions. A marker-assisted selection programme using the selected lines is currently under way.

## Supplementary material

Supplementary data can be found at *JXB* online.

Supplementary Table S1. Root hair length, density, and lateral root rating.

Supplementary Table S2. Descriptive trait statistics for parents (Aus276, MTU1010, and IR64) and mapping populations subjected to seedling-stage stress under direct-seeded upland conditions.

Supplementary Table S3. Descriptive trait statistics for parents (Aus276, IR64, and MTU1010) and mapping populations subjected to reproductive stress and non-stress treatments in lowland and direct-seeded upland conditions.

Supplementary Table S4. QTLs identified under different cultivation conditions in the Aus276/3*IR64 population.

Supplementary Table S5. QTLs identified under different cultivation conditions in the Aus276/3*MTU1010 population.

Supplementary Table S6. Percentage improvement of genotypes possessing QTLs under different conditions over lines not possessing QTLs for the Aus276/3*IR64 population.

Supplementary Table S7. Percentage improvement of genotypes possessing QTLs under different conditions over lines not possessing QTLs for the Aus276/3*MTU1010 population.

Supplementary Table S8. Broad-sense heritability of different nutrients in the Aus276/3*IR64 mapping population.

Supplementary Table S9. Status of the selected lines in the Aus276/3*IR64 and Aus276/3*MTU1010 populations.

Supplementary Table S10. The percentage improvement of lines possessing qGY10.1 and qDTF_10.1_ in the Aus276/3*IR64 population.

Supplementary Figure S1. Soil water potential measured by tensiometers at soil depths of 15 and 30cm (2013DUS).

Supplementary Figure S2. Soil water potential measured by tensiometers at soil depths of 30cm (2012DUR and 2013DUR, reproductive stage stress experiments).

Supplementary Figure S3. Major and epistatic QTLs for grain yield showing interaction between genomic locations on chromosomes 6, 8, and 9 in the Aus276/3*IR64 population.

Supplementary Figure S4. Distribution of genotypes showing gall infection on a gall rating scale of 1–5 in the Aus276/3*IR64 population (2013DUS).

Supplementary Data
