# Supplementary material for: Traits and QTLs for development of dry direct-seeded rainfed rice varieties
Source: J Exp Bot. 2014 Oct 21;66(1):225–44. doi: 10.1093/jxb/eru413 (PMC4265160; doi:10.1093/jxb/eru413)
Supplement: Supplementary Data [file supp_66_1_225__index.html]

Traits and QTLs for development of dry direct-seeded rainfed rice varieties — Traits and QTLs for development of dry direct-seeded rainfed rice varieties — Supplementary Data 

# Traits and QTLs for development of dry direct-seeded rainfed rice varieties

## Supplementary Data

Data files

**Files in this Data Supplement:**

- Supplementary Data - Supplementary Data
