# Supplementary material for: Traits and QTLs for development of dry direct-seeded rainfed rice varieties
Source: J Exp Bot. 2014 Oct 21;66(1):225–44. doi: 10.1093/jxb/eru413 (PMC4265160; doi:10.1093/jxb/eru413)
Supplement: Supplementary Data [file supp_eru413_jexbot120410_file001.pdf]

Table S1. *Root hair length, density and lateral root rating*

Root hair rating	Category
1	no root hair
2	short and sparse
3	short and dense
4	long and sparse
5	long and dense
Lateral root rating	
0	none or almost none
1	sparse
2	dense
3	dense and branching

Table S2. *Descriptive trait statistics for parents (Aus276, MTU1010 and IR64) and mapping populations subjected to seedling stage stress under direct seeded upland conditions*

Trait	Population	2012WUS	2013DUS
Seed weight (g)	Aus276	2.579± 0.26	2.72± 0.21
	MTU1010	1.849± 0.18	1. 43± 0.32
	Population mean	2.034± 0.015	2.11± 0.009
	Aus276	2.34± 0.24	2.12±0.25
	IR64	1.98± 0.13	1.66±0.14
	Population mean	2.07± 0.014	2.05±0.014
Dry shoot weight (g) (15 DAS)	Aus276	0.058± 0.019	0.146± 0.039
	MTU1010	0.062± 0.012	0.150± 0.091
	Population mean	0.061± 0.001	0.139± 0.002
	Aus276	0.071 ± 0.025	0.17±0.049
	IR64	0.067± 0.021	0.14±0.09
	Population mean	0.076±0.0014	0.15± 0.003
Dry shoot weight (g) (22 DAS)	Aus276	0.182± 0.025	0.272±0.032
	MTU1010	0.188± 0.063	0.230±0.020
	Population mean	0.198± 0.004	0.234±0.004
	Aus276	0.17±0.069	0.33±0.08
	IR64	0.19± 0.072	0.28± 0.02
	Population mean	0.20± 0.004	0.27±0.005
Dry shoot weight (g) (30 DAS)	Aus276	0.414± 0.17	0.465± 0.10
	MTU1010	0.355± 0.11	0.375± 0.14
	Population mean	0.401± 0.01	0.380±0.006
	Aus276	0.48± 0.018	0.49± 0.016
	IR64	0.44± 0.019	0.45± 0.016
	Population mean	0.51± 0.012	0.46± 0.009
First Emergence (Days to first emergence)	Aus276	-	3.0±0.41
	MTU1010	-	4.0± 0.56
	Population mean	-	3.6± 0.023
	Aus276	-	3.0±0.49
	IR64	-	4.1± 0.21
	Population mean	-	3.3± 0.03
Full emergence (Days to full emergence)	Aus276	-	7.0±0.80
	MTU1010	-	8.0±0.12
	Population mean	-	7.4±0.046
	Aus276	-	6.9±1.15
	IR64	-	7.8±1.01
	Population mean	-	6.5±0.07
RGR_{12} (g $days^{-1}$)	Aus276	0.167±0.05	0.0874±0.079
	MTU1010	0.152±0.01	0.0645± 0.038
	Population mean	0.196±0.003	0.0724± 0.002
	Aus276	0.126±0.35	0.096± 0.04
	IR64	0.151± 0.22	0.097± 0.01
	Population mean	0.166 ± 0.02	0.081± 0.002
RGR_{23} (g $days^{-1}$)	Aus276	0.116± 0.053	0.0763± 0.042
	MTU1010	0.092±0.034	0.0706± 0.019
	Population mean	0.137±0.003	0.0721± 0.002
	Aus276	0.178±0.50	0.058± 0.05
	IR64	0.160±0.44	0.068± 0.04
	Population mean	0.209± 0.03	0.073±0.003
RGR_{13} (g $days^{-1}$)	Aus276	0.283± 0.063	0.164± 0.048
	MTU1010	0.241± 0.033	0.135± 0.080
	Population mean	0.260± 0.004	0.144± 0.003
	Aus276	0.279±0.07	0.154± 0.050
	IR64	0.220±0.04	0.166± 0.011
	Population mean	0.246±0.004	0.175± 0.003
Nodal root number (15 DAS)	Aus276	10.4±1.16	7.8±2.29
	MTU1010	11.9±1.12	9.2±1.73
	Population mean	10.7±0.182	8.4±0.132

	Aus276	14.0±2.43	9.6±2.05
	IR64	8.0±2.31	9.0±1.11
	Population mean	10.4±0.14	10.52±0.12
Nodal root number (22 DAS)	Aus276	13.4±2.73	10.1±1.45
	MTU1010	10.1±1.18	9.3±1.60
	Population mean	12.6± 0.158	12.8±0.084
	Aus276	20.4± 3.01	13.0± 4.7
	IR64	13.0 ± 2.9	16.3± 2.22
	Population mean	13.1±0.17	18.4±0.27
Nodal root number (30 DAS)	Aus276	17.2±2.01	15±2.83
	MTU1010	15.4±1.19	17.6±1.28
	Population mean	23.9± 0.231	22.7±0.221
	Aus276	25.6± 3.97	22.0± 2.14
	IR64	20.7± 0.23	23.3± 4.9
	Population mean	18.0± 2.81	28.3± 0.29
Root hair length	Aus276	1.408± 0.32	1.467±0.43
	MTU1010	1.161± 0.30	1.133±0.76
	Population mean	1.243± 0.018	1.349±0.025
	Aus276	1.54±0.32	1.30± 0.35
	IR64	1.02±0.24	1.11± 0.11
	Population mean	1.30±0.019	1.27± 0.02
Root hair density	Aus276	1.446± 0.24	1.43±0.43
	MTU1010	1.322± 0.11	1.267±0.48
	Population mean	1.157± 0.014	1.32±0.025
	Aus276	1.36±0.29	1.39± 0.30
	IR64	1.09±0.11	1.16± 0.14
	Population mean	1.24±0.017	1.24± 0.018
Lateral root rating	Aus276	-	2.5±0.14
	MTU1010	-	1.8±0.33
	Population mean	-	2.2±0.021
	Aus276	-	2.1±0.22
	IR64	-	1.7±0.81
	Population mean		2.6±0.047
Biomass (g)	Aus276	55.34±9.09	45.56±3.97
	MTU1010	59.23±13.06	36.64±5.73
	Population mean	54.67±1.33	31.29±4.85
	Aus276	45.41±12.43	43.08± 9.56
	IR64	54.57± 8.26	26.88± 4.11
	Population mean	61.69±1.41	37.81± 5.46
Harvest index	Aus276	0.290± 0.064	0.170±0.072
	MTU1010	0.262± 0.021	0.139±0.016
	Population mean	0.274± 0.004	0.137±0.004
	Aus276	0.243±0.061	0.186±0.07
	IR64	0.296±0.065	0.102±0.02
	Population mean	0.232± 0.004	0.128±0.004
Grain yield (kg ha^{-1})	Aus276	561±88.83	680±198.25
	MTU1010	472+59.68	545±187.75
	Population mean	523± 92.58	770±201.23
	Aus276	861±100.01	962±211.02
	IR64	684±101.12	867± 119.7
	Population mean	766±80.59	886±121.74

Table S3. *Descriptive trait statistics for parents (Aus276, IR64, and MTU1010) and mapping populations subjected to reproductive stress and non-stress treatments in lowland and direct-seeded upland conditions*

Population	Trait		2012DUR	2013DUR	2012DUN	2012DLR	2012DLN
**Aus276/MTU1010**	GY (kg ha^{-1})	Aus276	1201	1098	2942	1305	3622
		MTU1010	643	522	3388	1172	3432
		Population mean	889	770	1662	1550	4244
		Highest line	1679	1267	3900	2846	6848
		Lowest line	129	122	265	588	1848
		$LSD_{0.05}$	504	683	1420	894	1542
	PHT (cm)	Aus276	92	69	95	83	96
		MTU1010	73	66	102	86	100
		Population mean	77	64	103	106	122
		Highest line	112	84	134	136	154
		Lowest line	48	41	70	70	81
		$LSD_{0.05}$	22	17	26	21	22
	DTF (days)	Aus276	75	93	78	80	80
		MTU1010	85	91	71	82	81
		Population mean	92	98	80	83	78
		Highest line	109	112	92	87	84
		Lowest line	72	80	67	71	72
		$LSD_{0.05}$	12	12	9	17	4
	EVV	Aus276	-	3.6	-	-	-
		MTU1010	-	4.7	-	-	-
		Population mean	-	4.9	-	-	-
		Highest line	-	2.4	-	-	-
		Lowest line	-	8.6	-	-	-
		$LSD_{0.05}$	-	2.4	-	-	-
	HI	Aus276	-	0.0098	-	-	-
		MTU1010	-	0.0069	-	-	-
		Population mean	-	0.0051	-	-	-
		Highest line	-	0.0302	-	-	-
		Lowest line	-	0.0014	-	-	-
		$LSD_{0.05}$	-	0.0017	-	-	-
**Aus276/IR64**	GY (kg ha^{-1})	Aus276	1696	961	2557	3346	4085
		IR64	593	492	1663	2368	3211
		Population mean	1073	717	1167	2563	4586
		Highest line	2152	1470	2770	5992	7378
		Lowest line	258	118	175	733	2182
		$LSD_{0.05}$	377	591	1073	1813	1636
	PHT (cm)	Aus276	87	70	96	104	98
		IR64	59	55	84	73	104
		Population mean	75	65	106	100	135
		Highest line	112	83	136	126	166
		Lowest line	50	42	70	65	74
		$LSD_{0.05}$	21	17	26	17	23
	DTF (days)	Aus276	81	88	75	81	80
		IR64	95	98	85	84	80
		Population mean	93	96	83	82	77
		Highest line	108	111	113	97	82
		Lowest line	72	88	71	71	68
		$LSD_{0.05}$	11	10	9	11	4
	EVV	Aus276	-	4.2	-	-	-
		MTU1010	-	5.6	-	-	-
		Population mean	-	5.0	-	-	-
		Highest line	-	1.5	-	-	-
		Lowest line	-	8.9	-	-	-
		$LSD_{0.05}$	-	2.6	-	-	-
	HI	Aus276	-	0.0076	-	-	-
		MTU1010	-	0.0030	-	-	-
		Population mean	-	0.0048	-	-	-
		Highest line	-	0.0220	-	-	-
		Lowest line	-	0.0010	-	-	-
		$LSD_{0.05}$	-	0.0013	-	-	-

Table S4. *QTL identified under different cultivation conditions in the Aus276/3*IR64 population.*

Trait	chr	Year	Flanking markers	Marker closest to Peak	Position	Additive effect	Additive effect %	R^2 (%)
GY	$qGY_{6.1}$	2012DUR	id6010515-id6015531	id6015531	107.5	-15.44	18.61%	10.1%
		2013DUR			104.5	-22.78	35.77%	11.2%
	$qGY_{8.1}$	2012DUR	id8003773-ud8001270	id8003773	56.3	-71.82	36.58%	10.4%
		2013DUR				-52.33	26.62%	8.2%
	$qGY_{9.2}$	2012DUR	id9004128-id9005890	id9004128	58.0	96.85	31.29%	7.4%
		2013DUR				42.39	44.44%	8.4%
	$qGY_{9.1}$	2012WUS	id9003600-id9004128	id9003600	49.6	30.09	15.71%	6.1%
		2013DUS				10.77	32.86%	6.1%
	$qGY_{10.1}$	2012DUN	id10005369-id10006378	id10006378	57.3	164.63	14.25%	7.0%
		2013DUN			58.7	183.31	8.0%	4.3%
		2012DLN			57.7	-289.61	6.28%	8.1%
		2012DLR			58.7	-361.22	13.32%	12.6%
	$qGY_{1.1}$	2013DUN	K_id1024836-id1026726	id1026726	162.3	-542.09	23.65%	7.6%
		2012DLN				-526.39	11.42%	8.1%
DTF	$qDTF_{6.1}$	2012DLN	ud6000218-id6004089	id6004089	26.4	-1.72	2.21%	12.6%
		2012DLR				-2.78	3.29%	12.2%
	$qDTF_{10.1}$	2012DUN	id10003870-id10005369	id10005369	45.7	-1.47	1.77%	7.6%
		2013DUN				-1.50	1.83%	8.5%
		2012DLN			49.7	-1.65	2.12%	14.4%
		2012DLR				-1.83	2.17%	9.5%
	$qDTF_{4.1}$	2013DUN	id4001205-id4002844	id4002844	13.2	-3.47	5.7%	4.9%
PHT	$qPHT_{1.2}$	2012DUN	K_id1024836-id1026726	id1026726	162.3	-6.51	6.06%	8.4%
		2013DUN				-4.28	3.8%	12.0%
	$qPHT_{1.1}$	2012DUN	K_id1021259-id1024167	id1024167	144.2	-4.22	3.68%	12.2%
		2013DUN				-4.70	4.18%	14.9%
		2012DLN				-3.62	2.62%	11.5%
		2012DLR				-4.87	4.92%	17.2%
		2012DUR	K_id1021259-id1024167	id1024167	142.2	-3.07	4.12%	7.7%
		2013DUR			142.2	-2.91	4.54%	9.6%
	$qPHT_{4.1}$	2012DUN	id4002844-id4004639	id4004639	20.2	1.83	1.58%	6.9%
		2013DUN				1.95	1.73%	6.2%
	$qPHT_{4.2}$	2012DUR	id4011542-id4012189	id4011542	121.6	-1.71	1.82%	6.4%
		2013DUR				-1.87	2.92%	6.8%
	$qPHT_{10.1}$	2012DLN	id10005369-id10006378	id10005369	52.3	-8.24	5.97%	12.8%
		2012DLR				-4.73	4.78%	12.5%

SW	$qSW_{5.1}$	2012DUR	id5004150-id5006615	id5006615	52.6	-0.11	5.4%	7.3%
		2013DUR				-0.12	5.6%	7.1%
NR	$qNR_{4.1}$	2012WUS	id4001205-id4002844	id4001205	14.2	1.10	5.23%	7.1%
		2013DUS			13.2	1.28	5.41%	6.6%
EVV	$qEVV_{9.1}$	2012WUS	id9003600-id9004128	id9003600	49.6	0.025	10.57%	5.8%
		2013DUS				0.034	21.93%	6.5%
RHL	$qRHL_{8.1}$	2012WUS	id8002306-id8003773	id8002306	46.2	-0.086	6.43%	7.1%
		2013DUS			43.3	-0.097	7.43%	6.8%
RHD	$qRHD_{8.1}$	2012WUS	id8000536-id8000845	id8000845	15.4	0.0617	5.1%	6.6%
		2013DUS				0.0627	5.2%	6.5%
	$qRHD_{5.1}$	2012WUS	id5004150-id5006615	id5004150	51.6	0.0885	7.3%	6.3%
		2013DUS				0.0984	8.1	6.6%
HI	$qHI_{6.1}$	2012WUS	ud6000218-id6004089	id6004089	30.4	0.024	11.60%	8.9%
		2013DUS			27.7	0.025	13.39%	6.2%
	$qHI_{10.1}$	2013DUN	id10005369-id10006378	id10006378	56.3	0.018	8.6%	7.3%
	$qHI_{10.2}$	2012WUS	id10006378-id10006890	id10006890	71.8	0.019	9.23%	7.7%
		2013DUS			71.3	0.027	17.87%	6.1%
Biomass	$qBio_{6.1}$	2012WUS	id6006288-id6010515	id6010515	59.6	-11.12	15.92%	8.1%
		2013DUS			61.6	-18.88	19.91%	4.6%
	$qBio_{10.1}$	2012WUS	id10003870-id10005369	id10005369	45.7	-6.58	10.37%	6.3%
		2013DUS			47.7	-8.78	9.14%	7.8%
Al conc	$qAl_{5.2}$	2013DUS	id5006615-id5007304	id5006615	63.3	-0.603	54.43%	3.0%
Cu conc	$qCu_{8.1}$	2013DUS	id8000845-id8001326	id8000845	29.3	0.005	10.36%	11.7%
Fe conc	$qFe_{5.2}$	2013DUS	id5006615-id5007304	id5006615	63.3	-0.644	38.26%	13.3%
N conc	$qN_{5.1}$	2013DUS	id5004150-id5006615	id5004150	51.6	-0.1180	12.46%	20.7%
P conc	$qP_{5.2}$	2013DUS	id5006615-id5007304	id5006615	63.3	-0.0615	23.3%	12.3%
Al uptake	$qAlU_{5.2}$	2013DUS	id5006615-id5007304	id5006615	63.3	-1.621	43.76%	11.5%
Fe Uptake	$qFeU_{5.2}$	2013DUS	id5006615-id5007304	id5006615	63.3	-4.481	78.49%	11.7%
P uptake	$qPU_{5.2}$	2013DUS	id5006615-id5007304	id5006615	63.3	-3.061	42.51%	12.3%
Zn uptake	$qZnU_{4.1}$	2013DUS	id4002844-id4004639	id4004639	20.2	0.1134	15.77%	12.0%
Nematode GR	$qGR_{5.2}$	2013DUS	id5006615-id5007304	id5006615	63.3	-0.9310	28.8%	13.7%
**Epistatic QTLs**								
**Trait**	**chr**	**Year**	**Flanking markers**	**Marker closest to Peak**	**Position**	**AA**	**AA %**	**R^2**
EVV	Chr 1	2013DUS	K_id1024836-id1026726	id1026726	158.3	0.032	20.62%	6.3%

	Chr 9		id9003600-id9004128	id9003600	49.6			
GY	Chr 6	2012DUR	id6010515-id601553	id6015531	107.5	52.98	43.86%	
	Chr 8		id8003773-ud8001270	id8003773	56.3			4.6%
	Chr 6		id6010515-id6015531	id6015531	107.5	-70.46	48.87%	
	Chr 9		id9004128-id9005890	id9005890	58.0			4.2%
	Chr 6	2013DUR	id6010515-id601553	id6015531	104.5	44.32	47.23%	
	Chr 8		id8003773-ud8001270	id8003773	56.3			5.1%
	Chr 6		id6010515-id6015531	id6015531	104.5	-39.55	44.14%	
	Chr 9		id9004128-id9005890	id9005890	58.0			6.5%
	Chr 4	2012WUS	id4008719-id4009390	id4008719	84.0	-2.89	14.69%	
	Chr 8		id8002306-id8003773	id8003773	52.2			7.0%
Seedling emergence	Chr 8	2013DUS	id8000845-id8001326	id8001326	25.3	-0.325	4.35%	
	Chr 9		id9006773-id9007315	id9007315	86.1			7.2%
RHD	Chr 5	2013DUS	id5004150-id5006615	id5006615	51.6	0.177	14.74%	
	Chr 7		id7005557-ud7002101	ud7002101	112.6			9.4%
DTFNS	Chr 4	2013DUN	id4004639-id4005212	id4004639	39.0	-1.20	1.45%	
	Chr 7		id7005557-ud7002101	ud7002101	108.6			7.3%
Fe conc	Chr 4	2013DUS	id4005570-id400683	id4005570	51.0	-326.03	70.03%	
	Chr 6		id6006288-id6010515	id6010515	68.6			11.7%
Mn conc	Chr 4	2013DUS	id4005570-id4006835	id4005570	51.0	-8.6689	18.49%	
	Chr 6		id6006288-id6010515	id6010515	69.6			11.9%
Fe uptake	Chr 4	2013DUS	id4005570-id400683	id4005570	51.0	-8371.9	64.64%	
	Chr 6		id6006288-id6010515	id6010515	68.6			11.7%
Mn uptake	Chr 4	2013DUS	id4005570-id4006835	id4005570	51.0	-265.76	20.96%	
	Chr 6		id6006288-id6010515	id6010515	69.6			16.9%

Abbreviated environment codes: *D = dry season, W = wet season, U = upland, L= lowland, N = non-stress (well-watered), R = reproductive-stage drought stress, S = seedling-stage drought stress*

Abbreviated observed traits: *GY: Grain Yield, DTF: Days to flowering, PHT: Plant height, SB: Shoot biomass, SW: Seed weight, NR: Nodal root number, RHL: Root hair length, RHD: Root hair density, EVV: Early vegetative vigor, HI: Harvest index, Al: Aluminum, Cu: Copper, Fe: Iron, N: Nitrogen, Mn: Manganese, P: Phosphorus, Zn: Zinc, GR: Gall rating, chr: Chromosome, AA: Additive x additive*

Table S5. *QTL identified under different cultivation conditions in the Aus276/3*MTU1010 population.*

Trait	chr	Year	Flanking markers	Marker closest to Peak	Position	Additive effect	Additive effect %	R^2
GY	$qGY_{1.2}$	2012DUN	id1000001-K_id1000955	id1000001	0.1	-231.40	11.7%	6.9%
		2013DUN				-234.77	8.3%	7.1%
		2012DUR				-44.09	20.6%	7.3%
		2013DUR				-15.41	21.8%	6.8%
	$qGY_{1.1}$	2012DLN	K_id1024973-id1026726	id1026726	167.7	-307.35	7.1%	9.3%
	$qGY_{6.2}$	2012DUR	id6006288-id6013772	id6006288	60.6	-58.30	26.8%	7.9%
		2013DUR				-48.82	20.2%	6.2%
	$qGY_{6.1}$	2012WUS	id6013772-id6015665	id6015665	105.1	-7.41	14.0 %	7.2%
		2013DUS				-8.89	17.1%	6.8%
		2012DLR	id6015665-ud6001296		108.3	142.99	8.7%	6.3%
	$qGY_{10.1}$	2012DUR	id10005474-id10006378	id10006378	61.2	-64.15	30.0%	8.9%
		2013DUR			60.0	-32.33	43.7%	10.0%
	$qGY_{11.1}$	2012DLR	id11001466-id11002436	id11002436	24.3	225.54	13.8%	8.0%
	$qGY_{11.2}$	2012DUN	id11009201-id11010385	id11010385	108.1	-61.63	8.7%	7.3%
		2013DUN				-37.39	6.8%	7.6%
DTF	$qDTF_{1.1}$	2012DUN	K_id1021259-id1024167	id1024167	145.2	4.4252	5.9%	6.9%
		2013DUN	id1024167-K_id1024973		149.8	3.3731	4.5%	6.6%
		2012DLN	K_id1021259-id1024167		143.2	0.7457	9.6%	7.5%
PHT	$qPHT_{1.1}$	2012DUN	id1024167-K_id1024973	id1024167	151.8	-4.0507	3.9%	8.2%
		2013DUN				-4.6728	4.1%	8.9%
		2012DUR				-2.5295	3.8%	6.6%
		2013DUR				-3.7866	5.9%	9.4%
		2012DLN				-6.3468	5.2%	9.9%
		2013DLR				-4.5307	4.3%	8.3%
	$qPHT_{1.3}$	2013DLR	id1005271-id1006691	id100527	42.5	-7.4538	7.0%	8.2%
NR	$qNR_{5.1}$	2012WUS	id5000759-id5001182	id5001182	10.3	0.9077	5.2%	6.7%
		2013DUS			12.3	0.9391	6.1%	6.6%
EVV	$qEVV_{9.1}$	2012WUS	id9002704-id9004128	id9004128	49.4	0.0189	8.9%	6.4%
		2013DUS				0.0136	9.3%	6.8%
RHL	$qRHL_{1.1}$	2012WUS	id1000001-K_id1000955	id1000001	0.1	0.0813	6.5%	6.6%
		2013DUS				0.0945	7.4%	6.9%
RHD	$qRHD_{1.1}$	2012WUS	id1005271-id1006691	id1005271	44.5	0.1194	9.8%	6.3%
		2013DUS				0.1331	8.2%	6.7%
Seedling	$qDTE_{9.1}$	2013DUS	ud9000737-id9002704	id9002704	40.4	0.1578	4.2%	6.6%

emergence								
Biomass	qBio$_{11.1}$	2012WUS	id11001466-id11002436	id11002436	25.3	-11.30	20.7%	7.9%
		2013DUS				-10.55	18.2%	7.3%
HI	qHI$_{11.1}$	2013DUN	id11001466-id11002436	id11002436	28.1	-6.13	10.2%	10.0%
	qHI$_{11.2}$	2013DUR	id6006288- id6013772	id6013772	85.5	-9.22	13.3%	8.2%
P conc (veg stage)	qPC$_{6.1}$	2012WUS	id6006288-id6013772	id6013772	95.6	-0.2125	17.4%	14.4%
		2013DUS			88.6	-0.3157	27.5%	13.5%
P uptake (veg stage)	qPU$_{4.1}$	2012WUS	id4008522-id4009900	id4008522	80.9	-0.4256	14.1%	16.1%
		2013DUS			81.9	-0.4211	13.8%	16.4%
**Epistatic QTLs**								
**Trait**	**chr**	**Year**	**Flanking markers**	**Marker closest to Peak**	**Position**	**AA effect**	**AA%**	**R^2**
P uptake (veg stage)	qPU$_{1.1}$	2012WUS	id1005271-id1006691	id1005271	45.5	-1.1654	38.6%	16.7%
	qPU$_{6.1}$	2013DUS	id6006288-id6013772	id6006288	59.6	-1.1597	38.1%	17.4%
DTF	qDTF$_{4.1}$	2012DUR	id4006835-id4008522	id4006835	66.6	2.0344	2.2%	5.7%
	qDTF$_{6.1}$		id6013772-id6015665	id6013772	104.1			3.0%
Dry shoot weight	qDSW$_{2.1}$	2012WUS	id2007378-id2007507	id2007507	72.6	0.0172	28.1%	
	qDSW$_{10.1}$		id10006378-id10006740	id10006740	67.8			10.8%

Abbreviated environment codes: *D = dry season, W = wet season, U = upland, L= lowland, N = non-stress (well-watered), R = reproductive-stage drought stress, S = seedling-stage drought stress*

Abbreviated observed traits: *GY: Grain Yield, DTF: Days to flowering, PHT: Plant height, HI: Harvest index, SW: Seed weight, NR: Nodal root number, RHL: Root hair length, RHD: Root hair density, EVV: Early vegetative vigor, P: Phosphorus, AA: Additive x additive*

Table S6. *Percentage improvement of genotypes possessing QTL (QTL +) under different conditions over lines not possessing QTLs (QTL -) for the Aus276/3*IR64 population.*

Aus276/3*IR64							
Trait	QTL	Experiment	Markers	QTL (+)	QTL (-)	IR64	Increase (%)
GY (kg ha^{-1})	qGY$_{6.1}$	2012DUR	id6010515	1195.64	1059.44	593	12.9%
			id6015531	1159.59	1002.23	593	15.7%
			id6010515+ id6015531	1197.14	1012.29	593	18.7%
		2013DUR	id6010515	821.56	741.48	492	10.8%
			id6015531	809.17	709.33	492	14.1%
			id6010515+ id6015531	839.32	713.80	492	17.6%
	qGY$_{8.1}$	2012DUR	id8003773	1268.48	1017.11	593	24.7%
			ud8001270	1230.14	1036.28	593	18.7%
			id8003773+ud8001270	1457.83	1154.09	593	26.3%
		2013DUR	id8003773	795.77	738.81	492	7.7%
			ud8001270	764.77	684.31	492	11.8%
			id8003773+ud8001270	852.50	712.84	492	19.6%
	qGY$_{9.2}$	2012DUR	id9004128	1169.25	1005.00	593	16.3%
			id9005890	1189.01	1095.09	593	8.6%
			id9004128+id9005890	1215.66	1017.34	593	19.5%
		2013DUR	id9004128	785.45	717.54	492	9.4%
			id9005890	814.51	758.47	492	7.4%
			id9004128+id9005890	850.61	766.65	492	11.0%
	qGY$_{9.1}$	2012WUS	id9003600	644.34	547.39	533	17.7%
			id9004128	561.54	453.44	533	23.8%
			id9003600+id9004128	711.32	533.23	533	33.4%
		2013DUS	id9003600	870.34	752.85	727	15.6%
			id9004128	756.57	650.85	727	16.3%
			id9003600+id9004128	925.33	749.13	727	23.5%
	qGY$_{10.1}$	2012DUN	id10006378	1756.12	1555.57	1663	12.9%
			id10005369	1683.05	1592.10	1663	5.7%
			id10006378+id10005369	1808.55	1579.09	1663	14.5%
		2013DUN	id10006378	2521.26	2343.66	1926	7.6%
			id10005369	2250.08	2131.14	1926	5.5%
			id10006378+id10005369	2429.04	2177.25	1926	11.6%
		2012DLN	id10006378	4498.38	4265.36	3211	5.4%
			id10005369	4573.57	4268.18	3211	7.1%
			id10006378+id10005369	4990.27	4487.97	3211	11.2%
		2012DLR	id10006378	2761.69	2465.60	2368	12.1%
			id10005369	2827.73	2422.45	2368	16.7%
			id10006378+id10005369	3241.93	2703.48	2368	19.9%
	qGY$_{1.1}$	2013DUN	K_id1024836	2441.99	2217.57	1926	10.1%
			id1026726	2332.11	2212.51	1926	5.4%
			K_id1024836+id1026726	2709.14	2402.37	1926	12.8%
		2012DLN	K_id1024836	4601.77	4367.01	3211	5.3%
			id1026726	4864.22	4295.79	3211	13.2%
			K_id1024836+id1026726	4980.83	4178.59	3211	19.2%
RHL	qRHL$_{8.1}$	2012WUS	id8002306	1.391	1.110	1.02	25.3%
			id8003773	1.410	1.198	1.02	17.5%
			id8002306+id8003773	1.580	1.090	1.02	44.9%
		2013DUS	id8002306	1.301	1.010	1.11	28.8%
			id8003773	1.376	1.145	1.11	20.2%
			id8002306+id8003773	1.430	1.040	1.11	37.5%
RHD	qRHD$_{6.1}$	2012WUS	id5004150	1.39	1.10	1.09	26.4%
			id5006615	1.30	1.09	1.09	19.3%
			id5004150+id5006615	1.46	1.06	1.09	37.7%
		2013DUS	id5004150	1.23	1.07	1.17	14.9%
			id5006615	1.24	1.04	1.17	18.2%

			id5004150+id5006615	1.27	1.02	1.17	24.5%
	$qRHD_{8.1}$	2012WUS	id8000536	1.25	1.09	1.09	22.5%
			id8000845	1.27	1.01	1.09	25.7%
			id8000536+id8000845	1.33	1.05	1.09	26.7%
		2013DUS	id8000536	1.38	1.17	1.17	17.9%
			id8000845	1.34	1.11	1.17	20.7%
			id8000536+id8000845	1.39	1.12	1.17	24.1%
NR	$qNR_{4.1}$	2012WUS	id4001205	24.86	21.63	20.7	15.1%
			id4002844	22.71	19.52	20.7	16.3%
			id4001205+id4002844	26.61	22.13	20.7	20.2%
		2013DUS	id4001205	23.93	21.21	23.3	12.8%
			id4002844	23.52	21.77	23.3	8.1%
			id4001205+id4002844	24.59	20.95	23.3	17.4%
EVV	$qEVV_{9.1}$	2012WUS	id9003600	0.233	0.203	0.220	14.8%
			id9004128	0.264	0.245	0.220	7.8%
			id9003600+id9004128	0.284	0.246	0.220	15.5%
		2013DUS	id9003600	0.172	0.155	0.166	10.9%
			id9004128	0.169	0.152	0.166	11.1%
			id9003600+id9004128	0.192	0.161	0.166	19.2%
P uptake (reproductive stage) (mg $plant^{-1}$)	$qPU_{5.2}$	2013DUS	id5006615	8.15	6.77	6.18	20.4%
			id500730	7.80	6.42	6.18	21.5%
			id5006615+id500730	9.21	7.44	6.18	23.8%
P conc (seedling stage) (mg)		2012WUS	id5006615	3.15	2.66	1.73	18.4%
			id500730	2.95	2.35	1.73	25.5%
			id5006615+id500730	3.23	2.54	1.73	27.1%
		2013DUS	id5006615	2.50	1.86	1.36	34.4%
			id500730	2.40	1.92	1.36	25.0%
			id5006615+id500730	2.80	1.99	1.36	40.7%
P conc (reproductive stage) (mg)		2013DUS	id5006615	0.282	0.241	0.250	17.1%
			id500730	0.270	0.232	0.250	16.4%
			id5006615+id500730	0.303	0.245	0.250	23.7%
Al uptake (µg $plant^{-1}$)	$qAlU_{5.2}$	2013DUS	id5006615	7034.31	6262.56	5762.1	12.3%
			id5007304	5845.47	4851.22	5762.1	20.5%
			id5006615+id500730	6351.48	5185.66	5762.1	22.5%
Al conc (µg)	$qAl_{5.2}$	2013DUS	id5006615	532.90	460.00	233	15,7%
			id5007304	447.11	395.79	233	13.2%%
			id5006615+id500730	772.22	638.00	233	21.1%
Fe uptake (µg $plant^{-1}$)	$qFeU_{5.2}$	2013DUS	id5006615	7163.13	6265.88	5673.1	14.3%
			id5007304	5753.01	5175.33	5673.1	11.2%
			id5006615+id500730	6461.20	5386.93	5673.1	19.9%
Fe conc (µg)	$qFe_{5.2}$	2013DUS	id5006615	516.90	467.00	229.4	10.5%
			id5007304	433.94	379.95	229.4	14.2%
			id5006615+id500730	736.89	633.00	229.4	16.3%
N uptake (mg $plant^{-1}$)	$qNU_{5.1}$	2013DUS	id5006615	24.60	21.08	23.80	16.7%
			id5007304	27.00	23.68	23.80	14.0%
			id5006615+id500730	21.30	17.47	23.80	21.9%
N conc (mg)	$qN_{5.1}$	2013DUS	id5006615	0.916	0.746	0.832	22.8%
			id5007304	0.852	0.733	0.832	16.2%
			id5006615+id500730	0.887	0.685	0.832	29.5%
Zn uptake (µg $plant^{-1}$)	$qZnU_{4.1}$	2013DUS	id4002844	754.17	676.23	670.18	11.5%
			id4004639	820.13	708.24	670.18	15.8%
			id4002844+id4004639	829.70	715.47	670.18	16.0%
Cu conc (mg)	$qCu_{8.1}$	2013DUS	id8000845	4.614	3.992	4.51	15.6%
			id8001326	4.829	4.141	4.51	16.6%
			id8000845+id8001326	5.083	4.275	4.51	18.9%
Nematode GR	$qGR_{5.2}$	2013DUS	id5006615	3.16	3.00	3.00	5.3%
			id5007304	3.00	2.89	3.00	3.8%

			id5006615+id5007304	3.89	3.33	3.00	16.8%

Abbreviated environment codes: *D = dry season, W = wet season, U = upland, L= lowland, N = non-stress (well-watered), R = reproductive-stage drought stress, S = seedling-stage drought stress*

Abbreviated observed traits: *GY: Grain yield, EVV: Early vegetative vigor, NR: Number of nodal root, RHL: Root hair length, RHD: Root hair density, P: Phosphorus, Al: Aluminum, N: Nitrogen, Fe: Iron, Zn: Zinc, Cu: Copper, Conc: concentration, GR: gall rating*

Table S7. *Percentage improvement of genotypes possessing QTL (QTL +) under different conditions over lines not possessing QTLs (QTL -) for the Aus276/3*MTU1010 population*

Aus276 x MTU1010							
Trait	QTL	Trial	Markers	QTL (+)	QTL (-)	MTU1010	Improvement (%)
GY (kg ha^{-1})	$qGY_{1.1}$	2012DUN	id1000001	3680	3285	3388	12.0%
			K_id1000955	3831	3509	3388	9.2%
			id100000+K_id1000955	3849	3339	3388	15.3%
		2013DUN	id1000001	4203	3866	4048	8.7%
			K_id1000955	4208	3922	4048	7.3%
			id100000+K_id1000955	4316	3724	4048	15.9%
		2012DUR	id1000001	806	711	643	13.4%
			K_id1000955	766	691	643	10.9%
			id100000+K_id1000955	928	775	643	19.5%
		2013DUR	id1000001	693	594	522	16.6%
			K_id1000955	651	585	522	11.3%
			id100000+K_id1000955	790	667	522	18.3%
	$qGY_{1.2}$	2012DLN	K_id1024973	4428	4037	3432	9.7%
			id1026726	4472	4098	3432	9.1%
			K_id1024973+id1026726	4582	4025	3432	13.8%
	$qGY_{6.1}$	2012DUR	id6006288	715	626	643	14.4%
			id6013772	747	644	643	16.1%
			id6006288+id6013772	857	694	643	23.6%
		2013DUR	id6006288	666	595	522	12.1%
			id6013772	807	721	522	12.0%
			id6006288+id6013772	846	714	522	18.5%
	$qGY_{6.2}$	2012WUS	id6013772	541	473	472	14.3%
			id6015665	471	418	472	12.7%
			id6013772+id6015665	538	458	472	17.5%
		2013DUS	id6013772	594	525	545	13.1%
			id6015665	564	512	545	10.2%
			id6013772+id6015665	683	577	545	18.3%
	$qGY_{10.1}$	2012DUR	id10005474	709	641	643	10.6%
			id10006378	722	632	643	14.3%
			id10005474+id10006378	882	740	643	19.2%
		2013DUR	id10005474	594	510	522	16.5%
			id10006378	513	458	522	11.9%
			id10005474+id10006378	740	608	522	21.7%
	$qGY_{11.1}$	2012DLR	id11001466	1533	1401	1172	9.4%
			id11002436	1903	1776	1172	7.1%
			id11001466+id11002436	2136	1827	1172	16.9%
EVV	$qEVV_{9.1}$	2012WUS	ud9000737	0.218	0.202	0.092	8.1%
			id9002704	0.208	0.197	0.092	5.7%
			ud9000737+id9002704	0.222	0.198	0.092	11.8%
		2013DUS	ud9000737	0.152	0.136	0.0706	11.7%
			id9002704	0.144	0.131	0.0706	10.1%
			ud9000737+id9002704	0.160	0.139	0.0706	15.7%
Seedling emergence	$qDTE_{9.1}$	2013DUS	ud9000737	3.71	3.83	4.0	3.2%
			id9002704	3.71	3.83	4.0	3.0%
			ud9000737+id9002704	3.43	3.79	4.0	9.5%
NR	$qNR_{5.1}$	2012WUS	id5000759	26.7	26	24	2.7%
			id5001182	27.4	25.7	24	6.8%
			id5000759+id5001182	28.1	24.6	24	14.2%
		2013DUS	id5000759	25.5	24.6	23	3.9%
			id5001182	25.1	24.2	23	3.9%
			id5000759+id5001182	26.8	24.0	23	11.7%
RHL	$qRHL_{1.1}$	2012WUS	id1000001	1.33	1.28	1.16	4.1%
			K_id1000955	1.57	1.40	1.16	12.3%

			id1000001+K_id1000955	1.71	1.46	1.16	17.3%
		2013DUS	id1000001	1.34	1.25	1.13	6.9%
			K_id1000955	1.50	1.36	1.13	10.1%
			id1000001+K_id1000955	1.70	1.49	1.13	13.9%
RHD	$qRHD_{1.1}$	2012WUS	id1005271	1.33	1.26	1.32	5.6%
			id1006691	1.40	1.25	1.32	12.4%
			id1005271+id1006691	1.50	1.26	1.32	19.2%
		2013DUS	id1005271	1.28	1.19	1.27	7.4%
			id1006691	1.22	1.17	1.27	4.3%
			id1005271+id1006691	1.29	1.16	1.27	11.4%

Abbreviated environment codes: *U = upland, L= lowland, D = dry season, W = wet season, N = non-stress (well-watered), R = reproductive-stage drought stress, S = seedling-stage drought stress*

Abbreviated observed traits: *GY: Grain yield, EVV: Early vegetative vigor, NR: Number of nodal root, RHL: Root hair length, RHD: Root hair density*

Table S8. *Broad-sense heritability (H) of different nutrients in Aus276/3*IR64 mapping population.*

Population	Trait	2013DUS
Aus276/3*IR64	Al concentration	0.48
	Ca concentration	0.42
	Cu concentration	0.72
	Mg concentration	0.43
	Fe concentration	0.44
	Mn concentration	0.1
	Na concentration	0.32
	S concentration	0.63
	Zn concentration	0.34
	Al uptake	0.86
	Ca uptake	0.21
	Cu uptake	0.51
	Mg uptake	0.21
	Fe uptake	0.88
	Mn uptake	0.18
	Na uptake	0.25
	S uptake	0.40
	Zn uptake	0.22

Table S9. *Status of the selected lines in Aus276/3*IR64 and Aus276/3*MTU1010 populations.*

S No.	Designation	QTLs
Aus276/3*IR64		
1	IR 94225-B-10-B	$qRHL_{8.1}$, $qGY_{6.1}$, $qGY_{8.1}$, $qGY_{9.1}$, $qGY_{9.2}$, $qGY_{10.1}$
2	IR 94225-B-82-B	$qNR_{4.1}$, $qRHD_{5.1}$, $qRHD_{8.1}$, $qGY_{6.1}$, $qGY_{8.1}$, $qGY_{10.1}$
3	IR 94225-B-201-B	$qNR_{4.1}$, $qRHD_{5.1}$, $qRHD_{8.1}$, $qRHL_{8.1}$, $qGY_{6.1}$, $qGY_{8.1}$, $qGY_{9.1}$,$qGY_{9.2}$, $qGY_{10.1}$
4	IR 94225-B-375-B	$qNR_{4.1}$, $qRHD_{5.1}$, $qRHD_{8.1}$, $qRHL_{8.1}$, $qGY_{6.1}$, $qGY_{8.1}$, $qGY_{9.1}$, $qGY_{9.2}$, $qGY_{10.1}$
5	IR 94225-B-394-B	$qNR_{4.1}$, $qRHD_{5.1}$, $qRHD_{8.1}$, $qRHL_{8.1}$, $qGY_{8.1}$, $qGY_{9.1}$, $qGY_{9.2}$, $qGY_{10.1}$
6	IR 94225-B-474-B	$qNR_{4.1}$, $qRHD_{5.1}$, $qRHL_{8.1}$, $qGY_{6.1}$, $qGY_{8.1}$, $qGY_{9.1}$, $qGY_{9.2}$, $qGY_{10.1}$
Aus276/3*MTU1010		
7	IR 94226-B-11-B	$qNR_{5.1,}$ $qEVV_{9.1}$, $qRHD_{1.1}$, $qGY_{6.1}$, $qGY_{10.1}$, $qGY_{1.1}$
8	IR 94226-B-15-B	$qNR_{5.1,}$ $qEVV_{9.1}$, $qRHD_{1.1}$, $qGY_{6.1}$, $qGY_{10.1}$, $qGY_{1.1}$
9	IR 94226-B-61-B	$qEVV_{9.1}$, $qRHL_{1.1}$, $qRHD_{1.1}$, $qGY_{6.1}$, $qGY_{10.1}$, $qGY_{1.1}$
10	IR 94226-B-177-B	$qNR_{5.1,}$ $qEVV_{9.1}$, $qRHD_{1.1}$, $qGY_{6.1}$, $qGY_{10.1}$, $qGY_{1.1}$
11	IR 94226-B-462-B	$qNR_{5.1,}$ $qEVV_{9.1}$, $qRHL_{1.1}$, $qRHD_{1.1}$, $qGY_{6.1}$, $qGY_{10.1}$, $qGY_{1.1}$
12	IR 94226-B-474-B	$qEVV_{9.1}$, $qRHD_{1.1}$, $qGY_{6.1}$, $qGY_{10.1}$, $qGY_{1.1}$

Table S10. *The percentage improvement of lines possessing $qGY_{10.1}$ and $qDTF_{10.1}$ in Aus276/3*IR64 population.*

Experiment	QTL	QTL (+)	QTL (-)	IR64	% improvement
2012DUN	$qGY_{10.1}$ + $qDTF_{10.1}$	1756.12	1555.57	1663	12.89
2012DLN	$qGY_{10.1}$ + $qDTF_{10.1}$	4498.38	4265.36	3211	5.46
2012DLR	$qGY_{10.1}$ + $qDTF_{10.1}$	2761.69	2465.6	2368	12.01
2012DUN	$qGY_{10.1}$ + $qDTF_{10.1}$	74	82	85	8 days
2012DLN	$qGY_{10.1}$ + $qDTF_{10.1}$	71	80	80	9 days

GY: Grain yield (Kg ha^{-1}), DTF: Days to flowering

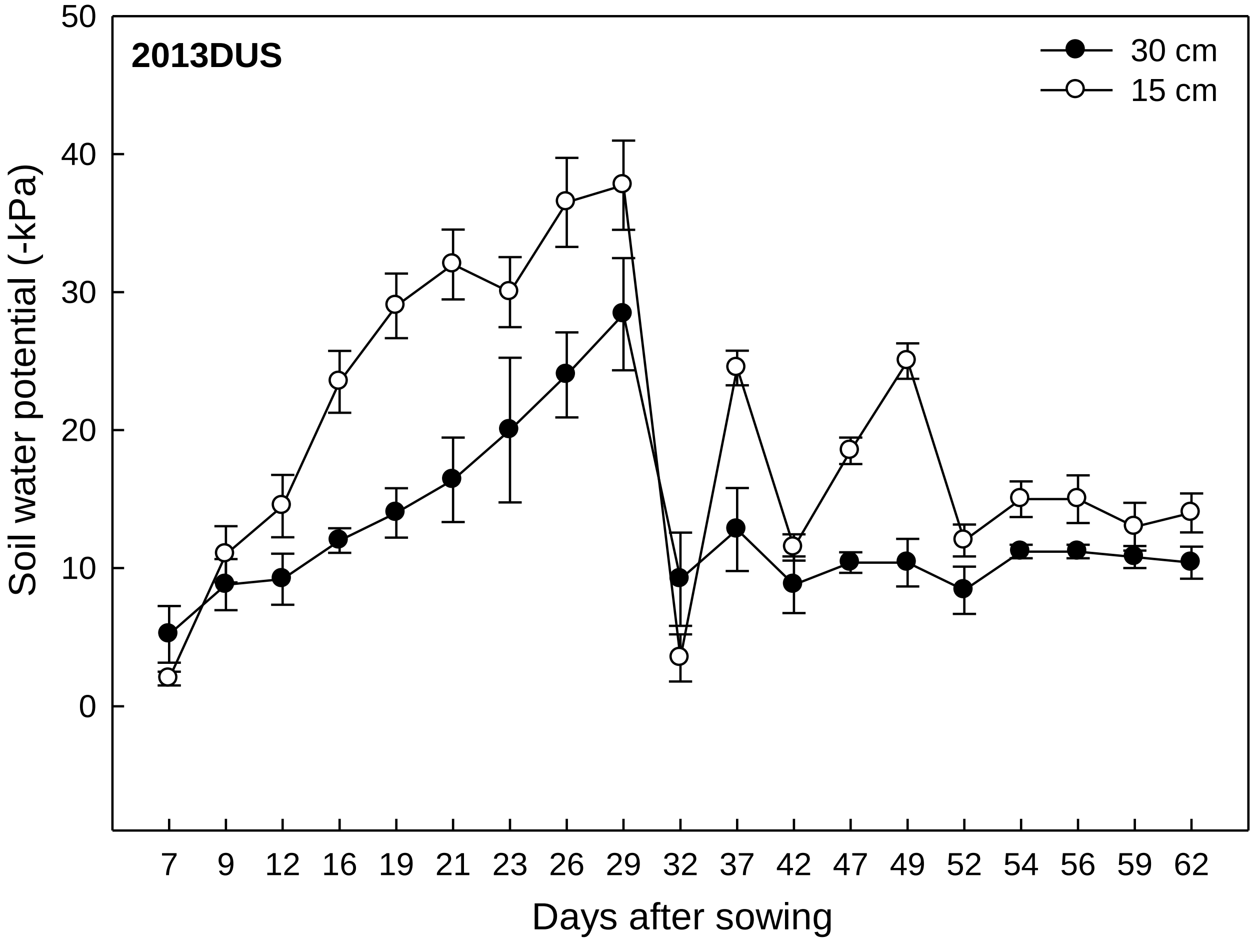

Figure S1. Soil water potential measured by tensiometers at soil depths of 15 and 30 cm (2013DUS). Values shown are mean +SE, n=3.

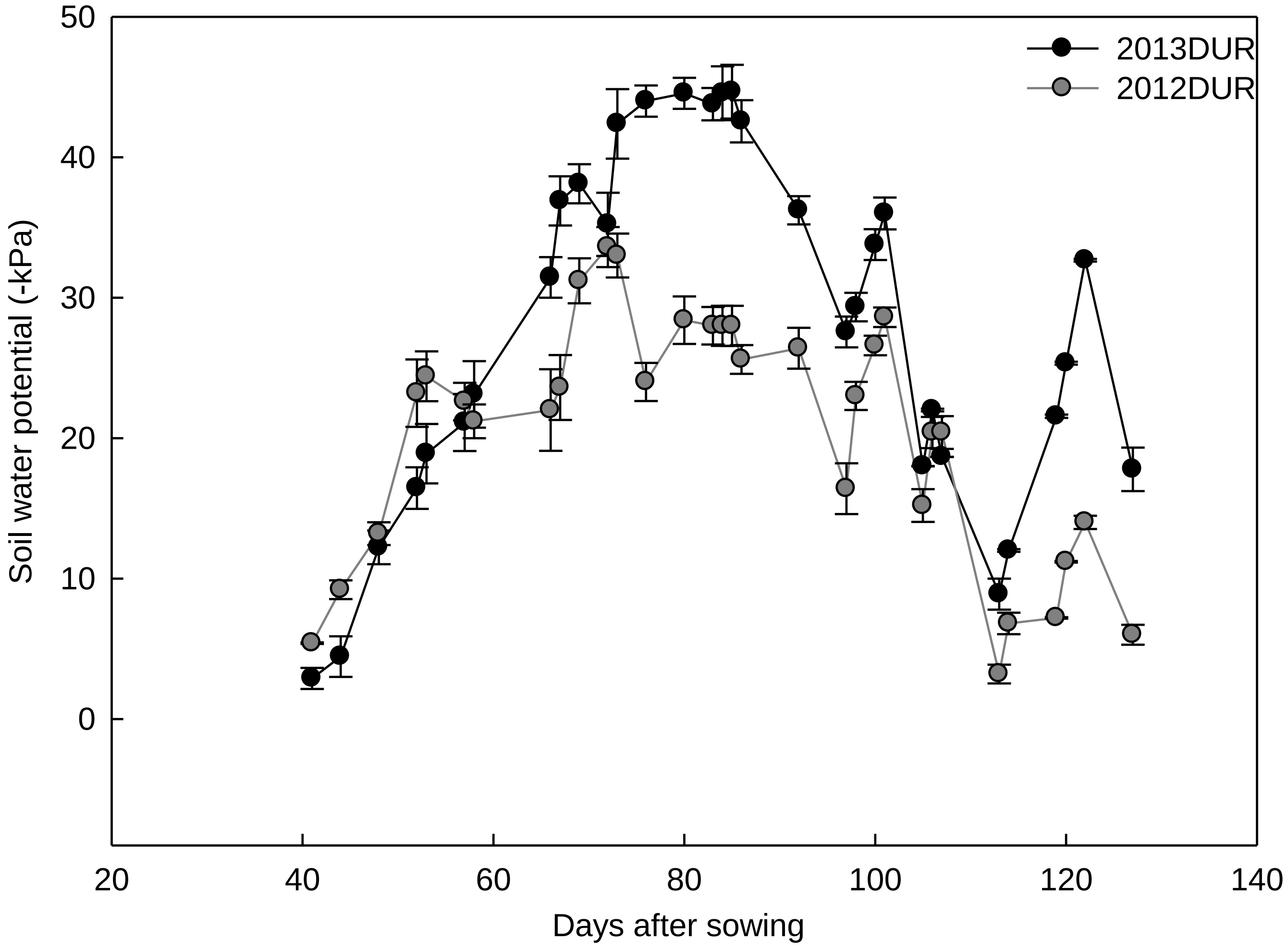

Figure S2. Soil water potential measured by tensiometers at soil depths of 30 cm (2012DUR and 2013DUR, Reproductive stage stress experiments). Values shown are mean +SE, n=3.

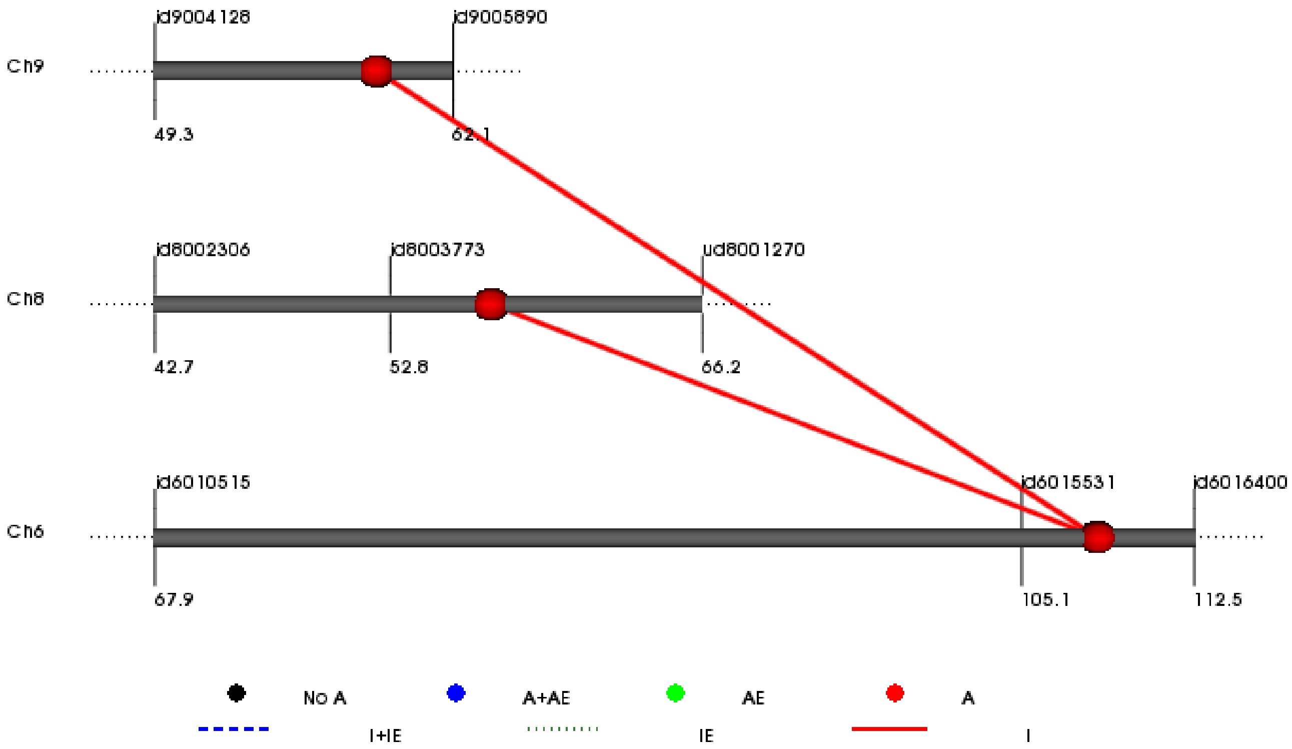

Figure S3. Major and epistatic QTLs for GY showing interaction between genomic locations on chromosome 6, 8 and 9 in the Aus276/3*IR64 population (A: Additive effect, I: Epistatic main effect, IE: Epistasis x Environment interaction, AE: Additive x Environment interaction).

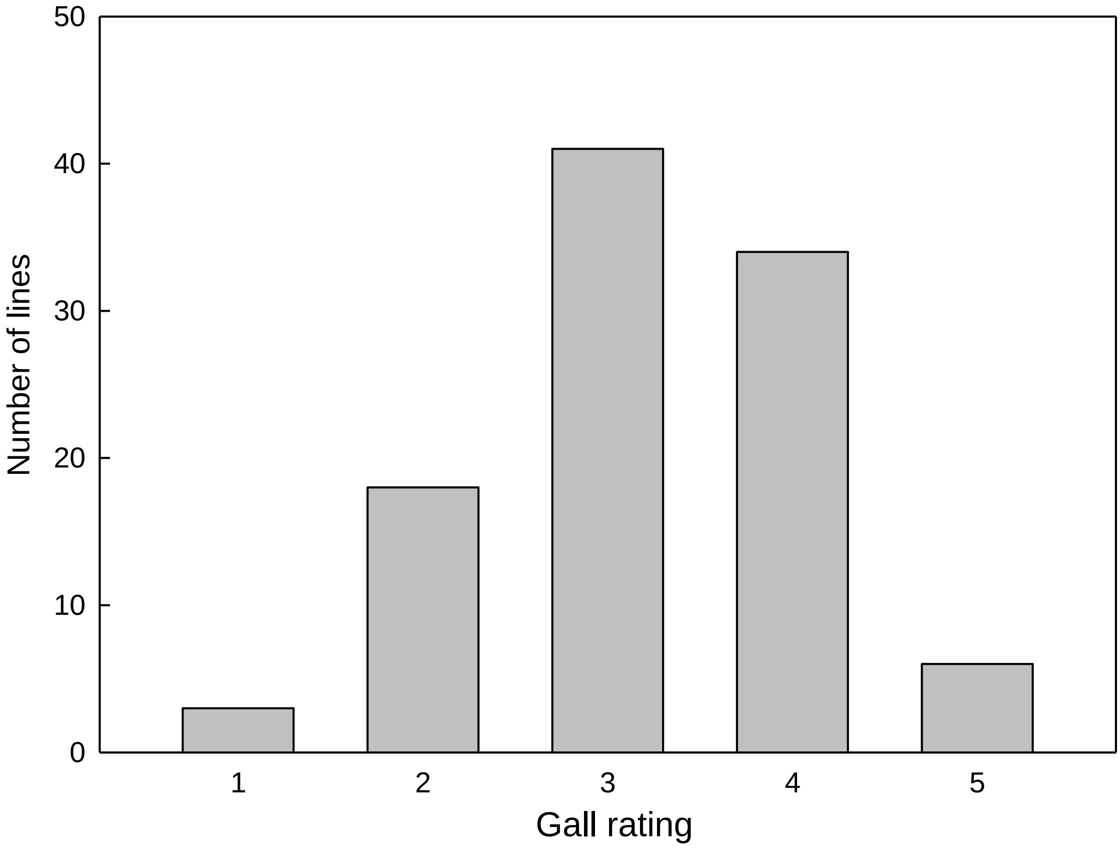

Figure S4. Distribution of genotypes showing gall infection on a gall rating scale of 1-5 in the Aus276/3*IR64 population (2013DUS).
